# Overexpression of *PaNAC03*, a stress induced NAC gene family transcription factor in Norway spruce leads to reduced flavonol biosynthesis and aberrant embryo development

**DOI:** 10.1186/s12870-016-0952-8

**Published:** 2017-01-06

**Authors:** Kerstin Dalman, Julia Johanna Wind, Miguel Nemesio-Gorriz, Almuth Hammerbacher, Karl Lundén, Ines Ezcurra, Malin Elfstrand

**Affiliations:** 1 Department of Forest Mycology and Plant Pathology, Uppsala Biocenter, Swedish University of Agricultural Sciences, Uppsala, Sweden; 2 KTH Biotechnology, Royal Institute of Technology, AlbaNova University Centre, Stockholm, Sweden; 3 Department of Biochemistry, Max Planck Institute for Chemical Ecology, Jena, Germany; 4 Department of Chemistry and Biotechnology, Uppsala Biocenter, Swedish University of Agricultural Sciences, Uppsala, Sweden; 5 Department of Microbiology, Forestry and Agricultural Biotechnology Institute, University of Pretoria, Pretoria, South Africa; 6 Department of Forest Mycology and Plant Pathology, SLU, PO. Box 7026, Uppsala, 75007 Sweden

**Keywords:** Bark, *Picea*, Transcriptome, NAC [for NAM (no apical meristem), ATAF (Arabidopsis *transcription* activation *factor)*, CUC (cup-shaped cotyledon)], Resistance to *Heterobasidion annosum*, ATAF1, Flavonoids, Leucoanthocyanidin reductase (LAR), Homeodomain proteins

## Abstract

**Background:**

The NAC family of transcription factors is one of the largest gene families of transcription factors in plants and the conifer NAC gene family is at least as large, or possibly larger, as in *Arabidopsis*. These transcription factors control both developmental and stress induced processes in plants. Yet, conifer NACs controlling stress induced processes has received relatively little attention. This study investigates NAC family transcription factors involved in the responses to the pathogen *Heterobasidion annosum* (Fr.) Bref. sensu lato.

**Results:**

The phylogeny and domain structure in the NAC proteins can be used to organize functional specificities, several well characterized stress-related NAC proteins are found in III-3 in *Arabidopsis* (Jensen et al. Biochem J 426:183–196, 2010). The Norway spruce genome contain seven genes with similarity to subgroup III-3 NACs. Based on the expression pattern *PaNAC03* was selected for detailed analyses. Norway spruce lines overexpressing *PaNAC03* exhibited aberrant embryo development in response to maturation initiation and 482 misregulated genes were identified in proliferating cultures. Three key genes in the flavonoid biosynthesis pathway: a *CHS,* a *F3’H* and *PaLAR3* were consistently down regulated in the overexpression lines*.* In accordance, the overexpression lines showed reduced levels of specific flavonoids, suggesting that *PaNAC03* act as a repressor of this pathway, possibly by directly interacting with the promoter of the repressed genes. However, transactivation studies of *Pa*NAC03 and *PaLAR3* in *Nicotiana benthamiana* showed that *Pa*NAC03 activated *PaLAR3A,* suggesting that PaNAC03 does not act as an independent negative regulator of flavan-3-ol production through direct interaction with the target flavonoid biosynthetic genes.

**Conclusions:**

*PaNAC03* and its orthologs form a sister group to well characterized stress-related angiosperm NAC genes and at least *PaNAC03* is responsive to biotic stress and appear to act in the control of defence associated secondary metabolite production.

**Electronic supplementary material:**

The online version of this article (doi:10.1186/s12870-016-0952-8) contains supplementary material, which is available to authorized users.

## Background

In plants, the NAC [for NAM (no apical meristem), ATAF (Arabidopsis *transcription* activation *factor)*, CUC (cup-shaped cotyledon)] family of transcription factors (TFs) is one of the largest plant TF gene families. The gene family is estimated to comprise 117 members in *Arabidopsis thaliana* and 144 and 161 respectively in rice and poplar [1, 2]. The NAC gene family in conifers appears to be at least as large as in *Arabidopsis* and might possibly even be expanded [3]. The boreal forest in the Northern hemisphere is dominated by conifers, many of which are economically and ecologically important. Still, relatively little is known about how conifers, and other gymnosperms, sense and respond to abiotic and biotic stress. General knowledge about inducible defence responses and their regulatory pathways are primarily derived from studies in angiosperm model plants, which in some cases can be extrapolated to gymnosperm systems [4–9], despite their evolutionary divergence [10]. A recent study showed that the accumulation of flavonoids and the gene induction pattern in the flavonoid pathway correlated to the level of resistance in Norway spruce to the root rot fungus *Heterobasidion annosum* (Fr.) Bref. sensu lato (hereafter referred to as *H. annosum* s.l.) [9]. *H. annosum* s.l. is a complex of five closely related species [11, 12] that have partly overlapping host ranges. These results indicated a differential control of defence responses between resistant and susceptible genotypes.

NAC TFs were first identified in forward genetic screens as key regulators of developmental processes [13–16]. NAC proteins have been shown to regulate central developmental processes such as embryo patterning and vascular patterning in both angiosperms and gymnosperms [15–18]. However, NAC proteins are also one of the most important groups of differentially regulated TFs in plant defence [19–21]. NAC TFs commonly possess a conserved DNA-binding NAC domain at the N-terminus, which includes nearly 160 amino acids that are divided into five subdomains (A-E) [22]. The C-terminal regions of NAC proteins are highly divergent [13, 22] and confer the regulatory specificity of transcriptional activation [1]. Based on the phylogeny of and domain structure in the NAC proteins it is possible to structure and organize the functional specificities of the conserved NAC domains and the divergent C-termini [1, 17, 22]. The NAC subgroups, e.g. subgroup III-3 in Arabidopsis, which contains the stress-related NAC proteins, ANAC019, ANAC055, ANAC072, ATAF1 and ATAF2, have common unique C-terminal motifs dominated by a negatively charged matrix with a few conserved bulky and hydrophobic amino acid residues that form the transactivation domains [1]. This group of paralogous Arabidopsis NAC genes show co-expression in response to stress hormones [20, 21, 23] and several members are known to act as regulators of plant responses to abiotic [19, 20, 23] and biotic [20, 24, 25] stressors. Transgenic plants overexpressing members of this subgroup (ATAF1, ATAF2, ANAC019 or ANAC055) show increased susceptibility to necrotrophic pathogens such as *Botrytis cinerea* or *Fusarium oxysporum* [20, 21, 24, 25] while an *anac019 anac055* double mutation [21] or expression of an ATAF1 repressor construct [24] lead to enhanced resistance against *B. cinerea.* Taken together, this suggests that subgroup III-3 NAC transcription factors may be important transcriptional integrators between biotic and abiotic stress. A number of NAC TFs with similarity to Arabidopsis subgroup III-3 NACs among the differentially regulated TFs in recent transcriptome studies of spruce responses to biotic stress [9, 26] indicate that spruce orthologs of well-characterized Arabidopsis NACs control similar programmes in spruce and Arabidopsis not only in plant development [17, 18] but also in plant responses to stress.

The aims of this study were to: I) analyse the classification and stress-induced expression pattern of *H. annosum* s.l.-induced Norway spruce NAC TFs; II) investigate the downstream target genes of *PaNAC03* in Norway spruce; III) investigate if *Pa*NAC03 had the capacity to regulate the promoter *PaLAR3*, a gene in the downstream regulation module. To address the first aim we queried sequence databases to identify homologous sequences, identified the modular structure and phylogenetic placement of *H. annosum* s.l.-induced Norway spruce NACs. We also determined the expression patterns of the *H. annosum* s.l.-induced NAC TFs in response to different stressors. To investigate downstream target genes of *PaNAC03* Norway spruce cell lines overexpressing *PaNAC03* were constructed and their transcriptome was compared with the wild-type Norway spruce cell line to identify misregulated genes. To address our last aim we isolated the promoter of *PaLAR3* and fused it to the GUS reporter gene and performed transactivation studies of *Pa*NAC03 and *PaLAR3* in *Nicotiana benthamiana*.

## Methods

### Sequence search and phylogeny

Six putatively unique transcripts (PUT) with similarity to angiosperm NAC transcription factors (Table 1) identified in previous RNAseq experiments [9, 26] were used to query the Norway spruce genome portal (http://congenie.org/) using Blastn [27] and TAIR (https://www.arabidopsis.org/) and Genbank using Blastx. The significant hits were downloaded and nucleotide and amino acid sequence alignments were made with *Picea* sequences from Genbank and *P. abies* 1.0 [3]. For phylogenetic analysis of the identified Norway spruce NAC genes additional Norway spruce gene models were downloaded from the Norway spruce genome portal and subgroup III-1, III-2 and III-3 Arabidopsis NAC amino acid sequences were downloaded from TAIR. The sequences were trimmed to the conserved N-terminal region and aligned with the Clustal W algorithm in MEGA 5.0 [28]. Phylogenetic trees were created using the Neighbor-joining algorithm in the same program with 1000 bootstrap values, p-distance estimations as a statistical model, uniform substitution rates and an estimation based on partial sequences with a cutoff value of 95%.

**Table 1:** Norway spruce subgroup III-3 NAC genes and their closest homolog in *Arabidopsis thaliana*

						TAIR	
Isogroup	Gene	Congenie (BlastN)	E-value	Best hit in NCBI	E-value	Locus	Annotation
isogroup00240^a^	PaNAC03	MA_8980g0010	0	ABK26029	0	AT1G01720.1	ATAF1
isogroup00812^b^	PaNAC04	MA_264971g0010	0	AAC32123	0	AT1G77450.1	ANAC032
isogroup02038^a^	PaNAC05	MA_5115g0010	0	ABK26029	1.00E-99	AT1G77450.1	ANAC032
isogroup05528^a^		MA_86256g0010	2.32E-144	ABK26029	2.00E-145	AT1G01720.1	ATAF1
		MA_64687g0010		ABK26029	2.00E-127	AT1G01720.1	ATAF1
		MA_75192g0010		ABK22535	0	AT4G27410.2	RD26
		MA_103386g0010		ABK26029	9.00E-145	AT1G01720.1	ATAF1
isogroup02925^b^		MA_8533126g0010	2.19E-111	ABR16510	5.00E-82	AT1G25580.1	SOG1
isogroup05889^b^		MA_23113g0010	1.83E-18	no hit		No hit	

^a^induced in both wounding and inoculation treatments

^b^induced only in response to inoculation treatment

Predicted subgroup III-3 Norway spruce NAC protein sequences were inspected for presence of a conserved N-terminal [22] and C-terminal domains [1]. The charge and hydrophobicity of the predicted proteins were estimated with EMBOSS *Pepinfo* software [29], the hydrophobicity of the predicted amino acid sequences was plotted using Kyte & Doolittles hydrophobicity index with a window of 11 amino acids. Sequence identity and similarity analysis of the full length and C-terminal regions of the identified Norway spruce NAC proteins was performed with the *ident* and *sim* functions of the Sequence manipulation suite [30].

### Determination of gene expression patterns

#### Biotic and abiotic stress

Thirty-year-old trees of eight independent Norway spruce genotypes which are part of a Swedish clonal forestry program and grow in a stand situated at Årdala, Sweden, (59°01’ N, 16°49’ E) [31] were inoculated with *H. annosum* s.l. The inoculation and sampling procedures are described in detail in Danielsson et al. [9]: Briefly, three ramets per genotype and two roots per ramet were used in the experiment. On one root, a wooden plug colonized by *H. annosum* s.s. (Sä 16–4) [32] was attached to an artificial wound on the root surface with Parafilm; the other root was wounded only and sealed with Parafilm. Phloem samples (ca 90 mm^2^ pieces) for RNA extraction were harvested at the start of the experiment (0 days post inoculation) and at 5 and 15 days post inoculation (dpi) and preserved in RNAlater (Ambion) for subsequent RNA extraction.

Total RNA was isolated according to Chang et al. [33]*.* Poly (A) + RNA was purified and amplified using MessageAmpIII (Ambion). Purified amplified RNA (aRNA, 1 μg) from each genotype were reverse transcribed with the iScript™ cDNA synthesis kit (Bio-Rad). The cDNA synthesis was diluted 1:1 in deionized water. Each genotype was used as an independent biological replicate.

#### Plant stress hormone treatments

To analyse the response of candidate genes to stress hormones and compare it to the response to *H. annosum* s.l., two-week-old Norway spruce seedlings (Rörby FP-65, 09 L022–1001) were transferred under axenic conditions to Petri plates with filter paper (five seedlings/plate), moistened with fertilized liquid media [34] and treated homogenized *Heterobasidion parviporum* (Rb175). For treatments with methyl jasmonate (MeJA) or methyl salicylate (MeSA) as previously described by Arnerup et al. [7]. Every treatment was performed in triplicate. After 72 h, seedlings were immediately frozen in liquid nitrogen and stored at −80 °C until further use. Total RNA was isolated according to Chang et al. [33] after DNAse I treatment one μg of total RNA was reverse transcribed with the iScript™ cDNA synthesis kit (Bio-Rad).

#### Somatic embryo maturation treatment

Samples for analysis of *PaNAC03* expression levels during embryo development, was a generous gift from Drs. Irena Molina and Malin Abrahamsson. Briefly, samples were collected from five sequential developmental stages (classification based on Zhu et al. [35]): +PGR (Proliferating cultures + Plant growth regulators (PGR) five days after subculture), —PGR (Proliferating cultures —PGR five days after subculture), EE (Early embryos differentiated after one week on maturation medium); LE1 and LE2 (late early embryos developed after two and three weeks on maturation medium, respectively). Three independent samples were collected for every stage and frozen in liquid nitrogen and stored at −80 °C until extraction. Total RNA were extracted with the Spectrum Plant Total RNA kit (Sigma Aldrich) after DNAse I treatment one μg of total RNA was reverse transcribed with the Quanta cDNA synthesis kit (Quanta Biosciences).

#### Quantitative reverse-transcribed PCR (qPCR)

For analyses of gene expression levels an aliquot of cDNA equivalent to 25 ng of RNA was used per 20 μL of PCR reaction using SSoFast EVAGreen Supermix (Bio-Rad) and a final concentration of 0.5 μM of each primer. Primers were designed using Primer3 software (http://primer3.wi.mit.edu/) with a melting temperature (Tm) between 58 °C and 60 °C, and amplicon length between 95 and 183 bp (Additional file 1). The thermal-cycling condition parameters, run on an iQ™5 Multicolor Real-Time PCR Detection System (Bio-Rad), were as follows: 95 °C for 30 s; 40 cycles of 95 °C for 5 s, 58 or 60 °C for 20 s. Each run was followed by a melt curve analysis to validate the specificity of the reaction. A linear plasmid standard curve was used to measure the PCR efficiency in each of the experiments, and primer pairs with efficiency lower than 95% were discarded. Two technical replicates were prepared for each sample.

The relative expression was calculated using the 2ΔΔCT-method [36, 37], transcript abundance was normalized to the reference genes *phosphoglucomutase* [38], *eukaryotic translation initiation factor 4A* (*elF4A*) [39] and *elongation factor 1-α* (*ELF1α*) [5]. The stability of reference gene expression was assessed with the Bestkeeper tool separately for every experiment [40]. Differential expression between treatments were tested with Kruskal-Wallis- and Mann–Whitney U-tests using the GraphPad Prism5 software (GraphPad Inc.).

### Transformation of Norway spruce

Full-length cDNA sequences of *PaNAC03* were obtained by amplification with the specific primers PaNAC03FL (Additional file 1), designed based on comparison of full-length or partial sequences of *P. abies, P. glauca* and *P. sitchensis* homologues, from a pool of cDNA from Norway spruce bark inoculated with *H. annosum* s.l. For the PCR reaction we used Dream-Taq Polymerase (Fermentas). AttB1 and attB2 adapters were added to the 1148 bp product by PCR using Dream-Taq Polymerase. The resulting PCR product was recombined into the pDONR/Zeo (Thermofisher) vector followed by LR recombination into pMDC32 vector [41]. The resulting vector was verified by test-digestion and sequencing.

Cell lines constitutively expressing *PaNAC03* were established by *Agrobacterium*-mediated transformation of Norway spruce somatic embryogenic cell line 95:61:21, as described by Minina et al. [42]. In brief, pMDC32:: PaNAC03 and pMDC32:: GUS [42] was transformed into the *Agrobacterium tumefaciens* C58C1 strain with the additional virulence plasmid pTOK47. Transformed bacteria were then grown overnight with the appropriate selection and collected by centrifugation and resuspended in infiltration buffer (10 mM MgCl2, 10 mM MES, pH 5.5, and 150 μM acetosyringone) to an OD_600_ of 10. Seven days old Norway spruce suspension cultures and *Agrobacterium* was mixed in a 5:1 ratio and acetosyringone was added to a final concentration of 150 μM. The co-cultivation was allowed to proceed for 4 h. Thereafter the cells were plated on a filter paper placed on the top of solidified proliferation medium with PGR [43] and incubated at room temperature in the darkness for 48 h. Then, filters were transferred on solidified proliferation medium with PGR containing 400 μg ml^−1^ timentin and 250 μg ml^−1^ cefotaxime and incubated under the same conditions for 5 days. Subsequently, filter papers were transferred onto fresh solidified proliferation medium with PGR containing 20 μg ml^−1^ hygromycin, 400 μg ml^−1^ timentin, and 250 μg ml^−1^ cefotaxime and subcultured onto fresh medium every week. The transgenic calli were picked from the plates after a month and transferred to solidified proliferation medium with PGR containing 20 μg ml^−1^ hygromycin, 400 μg ml^−1^ timentin, and 250 μg ml^−1^ cefotaxime. Transgenic lines were maintained on proliferation medium with PGR and 20 μg ml^−1^ hygromycin.

Nine transgenic lines were selected for DNA and RNA extraction for verification of the insert and expression levels respectively. To verify the transformation, DNA was extracted by homogenizing and boiling a 3–5 mm diameter callus in an Eppendorf tube in 20 μl 0.5 M sodium hydroxide at 95 °C, quickly centrifuging and diluting 5 μl of the supernatant in 495 μl 10 mM Tris–HCl pH 8. Five μl of the dilution was used in a 25 μl PCR reaction using DreamTaq (Thermo Scientific) and Hyg primers (Additional file 1).

Total RNA was extracted by using a modified CTAB extraction protocol [33]. After DNase I treatment (Sigma-Aldrich) cDNA was synthesised from 1 μg of total RNA using the iScript cDNA synthesis kit (BioRad). Expression levels of *PaNAC03* was tested by qRT-PCR by using an iQ5 Multicolor Real-Time PCR Detection System (BioRad) and SsoFast EvaGreen Supermix (BioRad) as stated previously and two independent lines (4.1 and 4.2) with expression levels 1.7 times higher than the WT cell line were selected for maturation initiation, RNA sequencing and chemical analysis. The initiation of somatic embryo maturation in the overexpression lines and the control line was done according to the protocol described by Filonova et al. [44], briefly for each line pre-weighed pieces of callus was placed on half strength LP medium for a week before the explants were transferred onto the maturation medium, the maturation response was scored after four and six weeks on maturation medium, embryos resembling the LE2, ME1 and ME2 stages [35] were noted.

#### Transcriptome profiling of PaNAC03 overexpression lines

##### RNA extraction and Illumina sequencing

The two selected overexpression (OE) lines, 4.1 and 4.2, along with the WT line (95:61:21) were incubated on solidified proliferation medium with PGR at room temperature in the darkness for six days and approx. 7 mm diameter large calli were picked from the lines and frozen in liquid nitrogen. The samples were ground in a mortar in liquid nitrogen and extracted by using the RNeasy Plant Mini Kit (Qiagen) using the RLT buffer and following the manufacturer’s instructions, thereafter the samples were treated with DNase I (Sigma-Aldrich). Three biological replicates per line were used for Illumina sequencing. The RNA integrity was analysed by using the Agilent RNA 6000 Nano kit (Agilent Technologies Inc.). Sequencing libraries were prepared at the SNP&SEQ Technology Platform (SciLifeLab, Uppsala) using the TruSeq stranded mRNA sample preparation kit according to the manual TruSeq stranded mRNA sample preparation guide. Sequencing was done using HiSeq 2500, paired-end 125 bp read length, v4 sequencing chemistry.

##### Filtering, mapping and differential expression

The raw sequences were filtered by a nesoni clip for the read pairs using *Nesoni* 0.128 (http://www.vicbioinformatics.com/nesoni-cookbook/index.html#) (See Additional file 2 for scripts used). To enable alignments to a reference database we constructed a Bowtie reference from the ‘Trinity contaminant free’ dataset downloaded from the Norway spruce genome portal (http://congenie.org/) using *Bowtie2* version 2.2.4 (http://bowtie-bio.sourceforge.net/bowtie2/index.shtml). The clipped read pairs were aligned to Trinity using *Tophat* version 2.0.13 [45]. The resulting alignment files from *Tophat* were provided to *cufflinks* version 2.2.1 to produce an assembly for each sample. The assemblies were then merged using *cuffmerge* (included in the cufflinks package). We then applied the newer workflow by running *cuffquant* (http://cole-trapnell-lab.github.io/cufflinks/manual/) that calculates transcript abundances from the single assembly file and the aligned read files produced by the *Tophat* run which was run separately for each sample. Differential expression analysis was performed with *cuffdiff* [45, 46].

#### Chemical analysis of Norway spruce overexpression lines

Norway spruce OE lines (4.1 and 4.2) overexpressing the *PaNAC03* gene and the wild-type cell line 95:61:21 were grown in liquid proliferation medium without PGR for two weeks. Thereafter, the cells were collected and flash frozen in liquid nitrogen after which the samples were freeze-dried. The freeze-dried samples were ground using a ball mill. Once pulverized, the sample-weight was noted. Specialised metabolite content was assessed with the method described by Hammerbacher et al. [47].

### Transactivation of p*Pa*LAR3 by *Pa*NAC03

#### PaLAR3 transactivation by PaNAC03

The *PaLAR3* promoter has two allelic forms, *PaLAR3A* and *PaLAR3B*. Both were amplified from genomic DNA using pPaLAR3A and pPaLAR3B primer sets (Additional files 1 and Additional file 3). After amplification, they were cloned into pJET1.2 plasmids using the CloneJET PCR cloning kit (Thermo scientific). From this plasmid, PCR products were amplified with the pPaLAR3A_2 and pPaLAR3B_2 primer sets (Additional file 1). These two PCR products were subsequently cloned into the destination plasmid pCF201 which was adapted from the pGA580 vector used for *Agrobacterium* transformation [48] by overlap extension PCR. To be able to do so, the destination plasmid was amplified into two separate PCR products. For the first PCR fragment the primers TetA2 forward and PUV5 reverse were used and for the second PCR fragment GUS forward and TetA2 reverse were used (Additional file 1). All the PCR product fragments were purified with the GeneJet PCR purification kit (Thermo Scientific) as instructed by the manufacturer’s protocol. The promoter fragments were separately combined with these destination fragments and amplified in a three fragment overlap extension PCR using the method from (Bryksin and Matsumura 2010) with the adaptation PCR protocol: Initial denaturation at 98 °C for 2 min, followed by three cycles of denaturation at 98 °C for 15 s, annealing at 60 °C for 2 min and elongation at 72 °C for 5 min, then 14 cycles of denaturation at 98 °C for 15 s, annealing at 60 °C for 30 s, elongation at 72 °C for 5 min, then the final elongation at 72 °C for 10 min. Single mutations (Additional file 3) in the *PaLAR3A* promoter were created by two fragment overlap extension PCR. Mut_XbaI_F or Mut_KpnI_F were combined with the TETA2_reverse primer to make the first fragment and Mut_XbaI_R or Mut_KpnI_R were combined with TetA2 forward for the second fragment. The two corresponding fragments were combined in an OEPCR with the same PCR conditions as described above. A double mutation was created by using Mut_XbaI_KpnI primers with the corresponding TETA2 primers and the same method was repeated.

The newly formed plasmids were isolated with *Dpn*I restriction endonuclease [49]. The restriction mix was incubated at 37 °C for 15 min and deactivated at 80 °C for 5 min. 1 μl of *DpnI* treated OE-PCR product was transformed into chemically component *E. coli* cells (One Shot® TOP10 Competent Cells, Invitrogen) and shake incubated for a minimum of 3 h at 37 °C. Colony PCR screen was performed with screening primers (Additional file 1). Positive clones were selected on agar plates with tetracycline (5 μg ml^−1^), and plasmids were isolated with the GeneJet Plamid Miniprep Kit (Thermo Scientific). Transformation of *Agrobacterium tumefaciens* (strain C58C1-RS with the helper plasmid pCH32) was done with the heat-thaw method as described [50]. Cells were plated on agar plates with tetracycline (5 μg ml^−1^), kanamycin (5 μg ml^−1^) and rifampicin (50 μl ml^−1^) and transformants were selected with colony PCR using the same primers as for *E.coli*.

The transactivation experiment is an adapted version of the one described in (Leborgne-Castel et al. 1999). Four to six weeks old *Nicotiana bethaminiana* plants were grown under a 16-h photoperiod at 23 °C. Infiltration occurred as described in (Voinnet et al. 2003). The following 1:1 mixes of *A. tumefaciens* harboring the different effector and reporter constructs were prepared. After 72 h, leaf disks were taken and GUS expression and total protein were measured. The GUS colorimetric assay was described in a protocol in Wilson et. al. [51] where 20 μl of cleared extract were added to 250 μl GUS assay buffer as well as to GUS assay buffer with 6 mM 4-Nitrophenyl β-D-glucuronide (PNPG). The reaction was incubated overnight covered in aluminum foil. OD_405nm_ was measured in a microplate reader of the type Fluostar Optima. The GUS activity was determined in mol PNP per minute and gram protein. The protein concentration was determined by the Bio-Rad protein assay [52]. Student t-tests were performed to calculate significant changes based on 6–12 biological replicates per measurement.

## Results

### Norway spruce contain multiple clade III-3NAC transcription factor gene family members

**Fig. 1:**
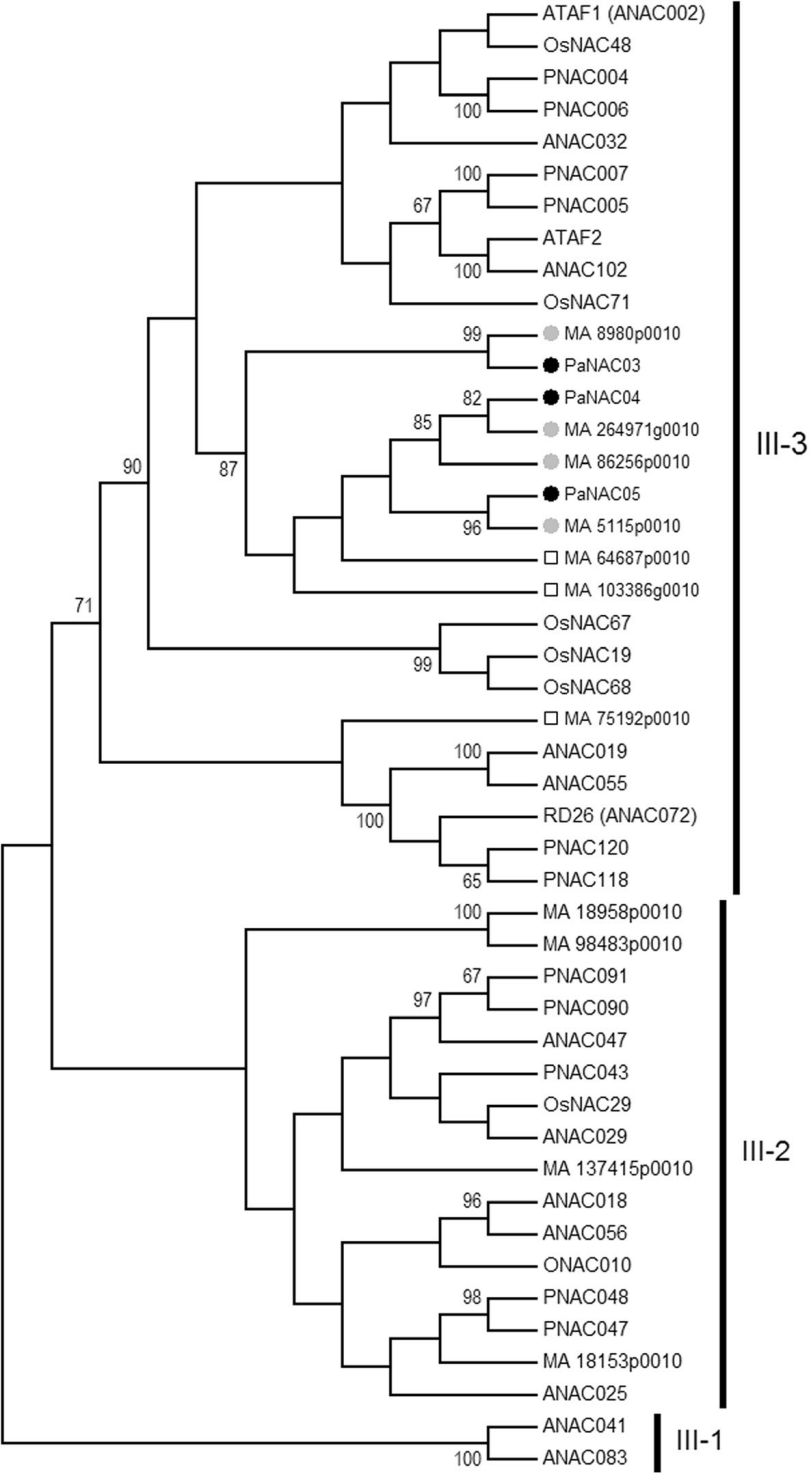
Neighbour-joining tree of subgroup III-1, 2 and 3 NAC family transcription factors in Norway spruce and Arabidopsis. Neighbour-joining tree based on the predicted amino acid sequence of the identified clade III-1, 2 and 3 NAC family transcription factors in Norway spruce gene models in *P.abies* 1.0 and the III-1, 2 and 3 NAC family transcription factors reported by Jensen and co-workers [1] namely AT1G77450.1 (ANAC032), AT1G01720.1 (ATAF1), AT5G63790 (ANAC102), AT5G08790 (ATAF2), AT4G27410.2 (RD26), AT1G52890 (ANAC019), AT3G15500 (ANAC055), AT1G61110 (ANAC025), AT3G15510 (ANAC056), AT1G52880 (ANAC018), AT2G33480 (ANAC041) and AT5G13180 (ANAC083). Poplar and rice sequences producing significant hits to Norway spruce clade III-3 NAC proteins: XP_002306280.1 (PNAC005), XP_002309945.1 (PNAC007), XP_002307447.1 (PNAC004), XP_002300972.1 (PaNAC006), XP_002305109.1 (PNAC043), XP_002305677.1 (PNAC048), XP_002316635.1 (PNAC047), XP_002319143.2 (PNAC090), XP_002325400.1 (PNAC091), XP_006387160.1 (PNAC120), XP_002316917.1 (PNAC118), XP_015645677.1 (ONAC010), XP_015630558.1 (OsNAC19/SNAC1), XP_015615093.1 (OsNAC29), XP_015620920.1 (OsNAC48), XP_015645028.1 (OsNAC67), XP_015623706.1 (OsNAC68) and XP_015617286.1 (OsNAC71). The Norway spruce sequences are represented by their gene model number. Black filled circles indicate subgroup III-3 Norway spruce genes for which there are both a gene model and a stress induced PUT available as indicated in the tree, grey filled circles indicate genes for which there exist only a partial PUT. Open squares indicate subgroup III-3 Norway spruce gene models for which there is no stress induced PUT available. Bootstrap values (1000 replications) are presented on the relevant nodes

The RNAseq dataset from the time course study of *H. annosum* s.s. inoculated Norway spruce [9, 26] contained six putatively unique transcripts (PUTs) with similarity to NAC TFs, all PUTs had at least one blastn hit in the *P. abies* genome v1.0 high confidence gene catalogue. Three of the PUTs, named *PaNAC03, PaNAC04* and *PaNAC05,* all had highly significant blastn hits to unique gene models in the *P. abies* v1.0 gene catalogue and significant blastx hits to Arabidopsis NACs (Table 1). *PaNAC03, PaNAC04* and *PaNAC05* all had homologs among clade III-3 NACs in Arabidopsis. A query of the *P. abies* genome v1.0 gene catalogue and a phylogenetic analysis of Norway spruce, rice, poplar and Arabidopsis protein sequences show that the Norway spruce genome has at least seven NAC gene models (Fig. 1) which fall within subgroup III-3 described by Jensen et al. [1]. We essentially see four clades within subgroup III-3, the predicted amino acid sequence of six of these genes, including *PaNAC03- PaNAC05,* form a sister group to a clade with members from all angiosperm species including ANAC032, ATAF1, ATAF2, ANAC102. The Norway spruce clade and two other clades, one of them specific to rice, are distinctly separated from the ANAC019, ANAC055, ANAC072, PNAC118 and PNAC120 protein sequences (Fig. 1). The six sequences in the Norway spruce clade share a higher amino acid similarity with each other than with MA_75192p0010, which clusters closer to the ANAC019, ANAC055, ANAC072, PNAC118 and PNAC120 branch (Additional file 4 and Additional file 5).

*PaNAC03* (MA_8980g0010), *PaNAC04* (MA_264971g0010), and *PaNAC05* (MA_5115g0010) correspond to isogroup00240, isogroup00812 and isogroup02038 respectively (Table 1) identified in the time course study of the Norway spruce’s transcriptional responses to *H. annosum* s.s. [9, 26]. The predicted proteins from *PaNAC03* and *PaNAC04* share a maximum of 81% identity and 90% similarity in the conserved N- terminal domains and 59% similarity over the complete predicted protein sequence (Additional file 5). The two sequences cluster closely in the phylogeny together with three other potential NAC genes, all highly similar (Additional file 5). The third expressed Norway spruce clade III-3 like NAC, PaNAC05, clusters outside this group of highly similar NAC sequences (Fig. 1) and the protein share approximately 40% identity on amino acid level with the PaNAC03 and PaNAC04 proteins.

The conserved N-terminal A-E motifs [22] were present in all the identified Norway spruce NACs (Additional file 4). The C- terminal region is highly conserved between PaNAC04, MA_103386p0010 and MA_86256p0010 and is dominated by polar and charged amino acids (Additional file 4). PaNAC03 share a common C-terminal motif (SEKEE (V/I) QSSFRLE, Additional file 4) with all Norway spruce clade III-3 NACs except PaNAC05*.* The C- terminal motifs in Norway spruce subgroup III-3 NACs are different from the negatively charged matrix with a few conserved bulky and hydrophobic amino acid residues in *Arabidopsis* subgroup III-3 NACs [1].

### Pathogen-induced expression of clade III-3-like Norway spruce NACs

**Fig. 2:**
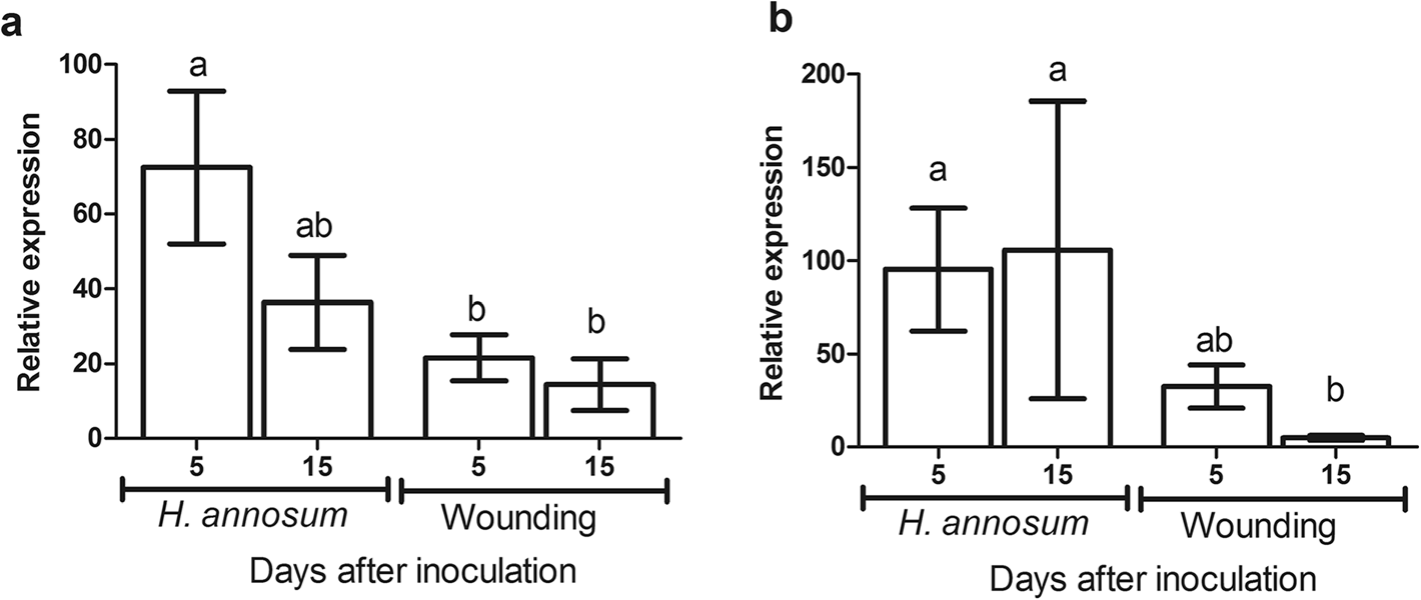
Expression pattern of NAC genes in bark of mature Norway spruce trees. The relative expression levels over the control, determined with qRT-PCR, of (**a**) *PaNAC03* (isotig01210) and (**b**) *PaNAC04* (isotig02452) in response to wounding and inoculation with *H. annosum* s.s. at 5 and 15 days after inoculation. The standard error (SE) is shown for time point and treatment. Superscript letters indicate significant differences between treatments (One-way ANOVA, Tukey’s post test) (*N =* 7)

**Fig. 3:**
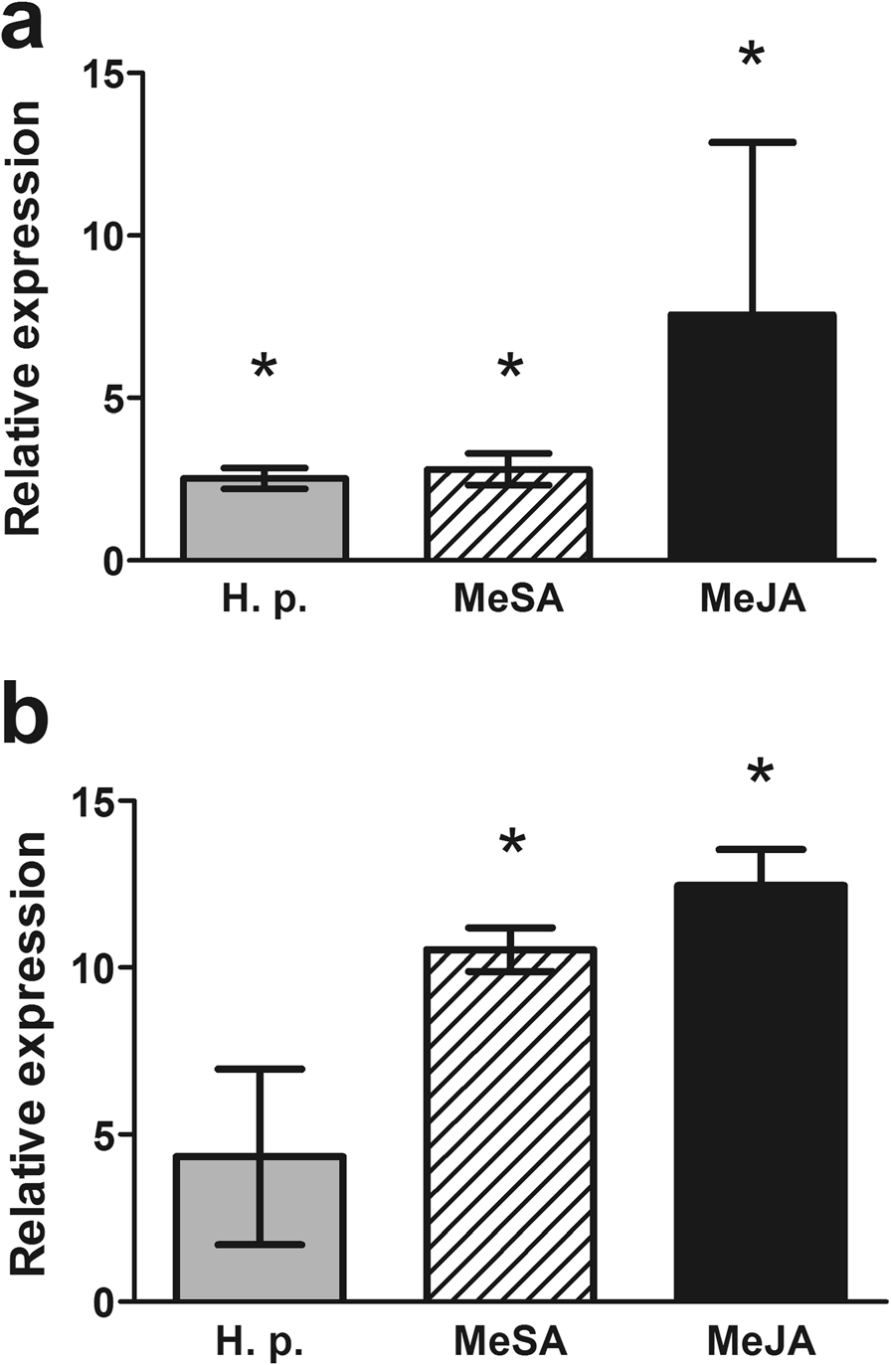
Expression patterns of subgroup III-3 NAC genes in response to *Heterobasidion*-induction, MeSA and MeJA treatments in seedlings. The relative expression levels over the control, determined with qRT-PCR, of (**a**) *PaNAC03* and (**b**) *PaNAC04*; in response to inoculation with *H. parviporum* (H.p.), MeSA and MeJA. The bars indicate standard error (SE) and asterisks indicate *P <*0.05 (Mann–Whitney U test)

We selected *PaNAC03* and *PaNAC04* for expression analysis as representatives of NACs responding to both wounding and inoculation (*PaNAC03*) and of NACs primarily responding to inoculation (*PaNAC04*) in the time course study of Norway spruce transcriptional responses to *H. annosum* s.s. [9, 26], as these PUTs were the most highly expressed in either category. The qRT-PCR analysis showed that *PaNAC03* is significantly induced in response to both inoculation and wounding treatments (*P* <0.05 for both treatments) compared to the control although the induction level was significantly higher after inoculation compared to wounding at 5 dpi (*P* = 0.01) (Fig. 2a). *PaNAC04* was significantly induced at 5 dpi both after wounding and inoculation with *H. annosum* s.s. (*P* =0.008 and *P* = 0.004 respectively) compared to the control. The qRT-PCR data also showed that *PaNAC04* transcript levels were significantly higher after inoculation compared to the wounding treatment at 15 dpi (*P* = 0.02) (Fig. 2b). The responsiveness of *PaNAC03* and *PaNAC04* to *H. parviporum* inoculation or to plant defence hormones (MeJA and MeSA) was tested in young seedlings. Both genes were significantly induced in response to MeJA and MeSA treatments (Figs. 3a and b) but only *PaNAC03* was significantly induced in response to fungal inoculation (Fig. 3a).

### *PaNAC03* overexpression in Norway spruce leads to altered developmental and metabolite profiles

#### PaNAC03 overexpression lines show abnormal embryo development

**Fig. 4:**
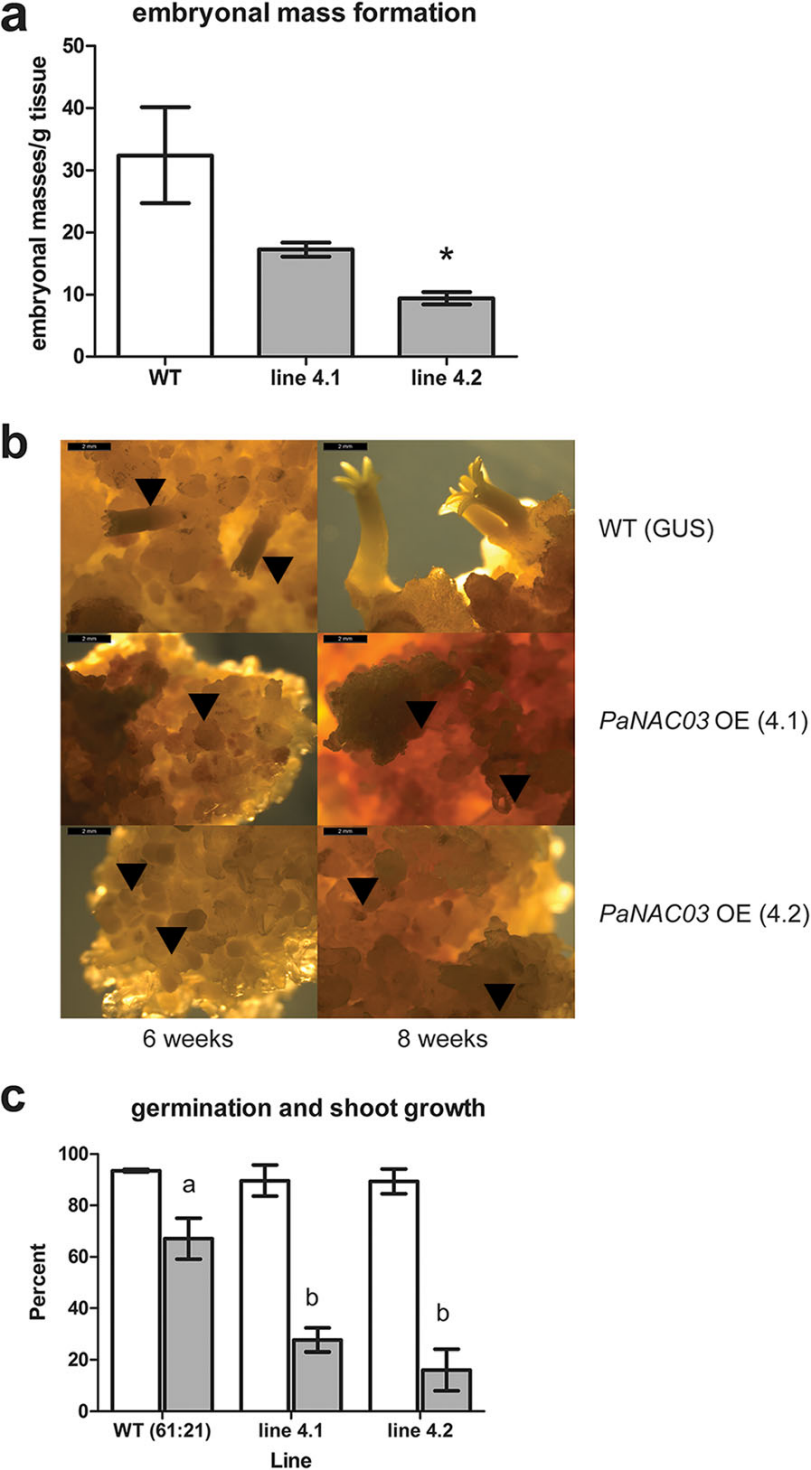
*PaNAC03* overexpression lines lack normal protoderm and display a disturbed maturation response. Embryonal mass formation per gram of proliferating tissue, the asterisk indicate significantly differences in embryonal mass formation between control (*P <*0.05 t-test) (**a**). Photos taken after six and eight weeks (**b**) on maturation medium for the overexpression lines PaNAC03_4.1 and PaNAC03_4.2, expressing *PaNAC03* at equal levels (1.7 times the WT), and the wild type represented by the pMDC32-GUS transformed WT line 95:61:21. Scale bar corresponds to 2 mm. Black arrowheads indicate developing embryos. Germination (open bars) and epicotyl growth (grey bars) one and two months after transfer to germination medium (**c**) in WT and the two OE lines, superscript letters indicate significant differences between treatments (One-way ANOVA, Tukey’s post test)

Eight selected hygromycin-resistant lines were verified to be transformed with pMDC32:: PaNAC03. Five of these lines were shown to moderately overexpress *PaNAC03*, 1.2-2.2 times the WT line (Additional file 6). In the WT line, *PaNAC03* expression is at, or below, the detection limit during early embryo development and no truly quantifiable expression was detected until LE2 (3 weeks after ABA treatment) (Additional file 7). Two OE lines, 4.1 and 4.2, expressing *PaNAC03* at equal levels (1.7 times compared to WT) were tested for maturation capacity with a standard maturation protocol [44] (Filonova et al. 2000). Both lines formed distinct embryonal masses in response to ABA treatment albeit at a lower frequency than the WT line (t-test, P = 0.095 and P = 0.048 for OE line 4.1 and 4.2 respectively, Fig. 4a). However, the embryonal masses appeared to lack a normal protoderm and rarely developed into normal mature embryos (Fig. 4b). Thus, the proliferating OE lines 4.1 and 4.2 and the WT, were selected for transcriptome and metabolite profiling (Additional file 6). A small number of mature embryos with a reduced number of/or fused cotyledons, were obtained from the OE lines (Fig. 4c). The embryos from the OE lines showed a normal germination response after a standard desiccation treatment [44], but a significantly smaller fraction of the germinated embryos showed epicotyl formation and growth (Fig. 4c).

#### A limited number of consistently misregulated genes are found in the overexpression lines

The transcriptomes of the *PaNAC03* OE lines and the WT line were sequenced with Illumina HiSeq sequencing generating 15.6-17.9 M reads per sample that passed Illumina’s chastity filter and between 15.4 and 17.8 M read pairs were kept after *Nesoni* filtering (Additional file 8). The overall read mapping rate from *Tophat* was 28–63% where most samples had around 60% mapping (Additional file 9).

**Fig. 5:**
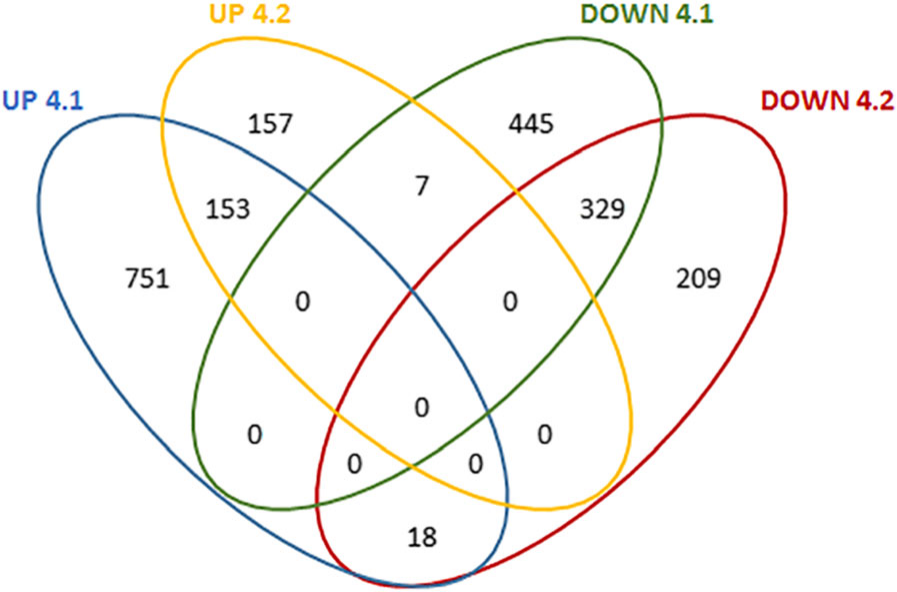
Venn diagram identifying the 482 consistently misregulated Norway spruce gene models in *PaNAC03* OE-lines. Up 4.2 (yellow line) are the upregulated genes in *PaNAC03* OE-line 4.2, Up 4.1 (blue line) are the upregulated genes in *PaNAC03* OE-line 4.1 and the intercept between them comprises the 153 consistently upregulated genes. Similarly Down 4.2 (red line) are the downregulated genes in OE-line 4.2 and Down 4.1 (green line) are the downregulated genes in OE-line 4.1 and the intercept between them comprises the 329 consistently downregulated genes

The analysis of the RNA-seq data-set showed that compared to the WT line 4.1 and 4.2 had 1683 and 740 differentially regulated genes respectively, and 482 genes were consistently misregulated in both OE-lines (Fig. 5). Of these, 153 were consistently up-regulated in both 4.1 and 4.2 and 329 were consistently down-regulated in both OE lines (Fig. 5). The down-stream analyses of the transcriptome data focussed on these consistently misregulated genes to understand the impact of *PaNAC03* OE on Norway spruce gene expression patterns.

A Fischer exact test (FDR <0.05) of the GO terms associated with the genes consistently upregulated in *PaNAC03* OE lines indicated that genes associated with the gene ontology (GO) categories such as cell wall macromolecule biosynthetic process (GO:0044038), carbohydrate metabolic process (GO:0005975), hemicellulose metabolic process (GO:0010410) and developmental process (GO:0032502) were overrepresented among the consistently up-regulated genes (Additional file 10) compared to the dataset as a whole. Two of the five most highly upregulated gene models encode homeodomain proteins, MA_122121g0010 and MA_114226g0010, which are potentially connected to developmental patterning in Norway spruce (Additional file 11) a third homeodomain protein, MA_10427484g0010, was also found among the consistently upregulated genes. MA_122121p0010 is related to *PaHB2* and the *Arabidopsis* gene *GLABRA2* [53–55] and was the most strongly and consistently upregulated gene model, as it was upregulated approximately 45 times compared to wild type. MA_114226g0010 encodes a protein with very high similarity to *Pa*KN4 (AAV64000).

The consistently down regulated genes in *PaNAC03* OE lines associated with the GO categories: protein folding (GO:0006457), metabolic process (GO:0008152), response to light stimulus (GO:0009416), response to abiotic stimulus (GO:0009628), response to stress (GO:0006950) and response to hydrogen peroxide (GO:0042542) (Fischer exact test FDR <0.05; Additional file 10). Again, the most strongly regulated gene in the consistently down regulated domain was a gene model, MA_10251997g0010, with similarity to the *Arabidopsis* transcription factor *KANADI* (AT5G16560.1) (Additional file 12). Four peroxidases associated with the GO term GO:0042542 were down regulated in the OE lines, three of these were class III peroxidases MA_195910g0010 (PabPrx132), MA_195775g0010 (PabPrx131) and MA_185755g0010 (PabPrx01) (Additional file 11).

#### PaNAC03 overexpression lines show reduced levels of flavanoids

Interestingly, three key genes in the flavonoid biosynthesis pathway were concomitantly down-regulated in the *PaNAC03* OE-lines: a *chalcone synthase,* MA_10359605g0010, homologous to the *Arabidopsis* gene *transparent testa 4* (*TT4*, AT5G13930), a *flavonoid 3’-hydroxylase* (*F3’H*, MA_10434709g0010) a possible homologue to the *Arabidopsis* gene *transparent testa 7* (*TT7*, AT5G07990) and the previously described *PaLAR3* gene (MA_10001337g0010) [47, 56] (Additional file 12). We only detected one consistently induced gene associated within the phenylpropanoid pathway, MA_10429470g0020, which encodes an *isoflavone reductase* with similarity to AT4G39230 which might be involved in lignin biosynthesis.

**Fig. 6:**
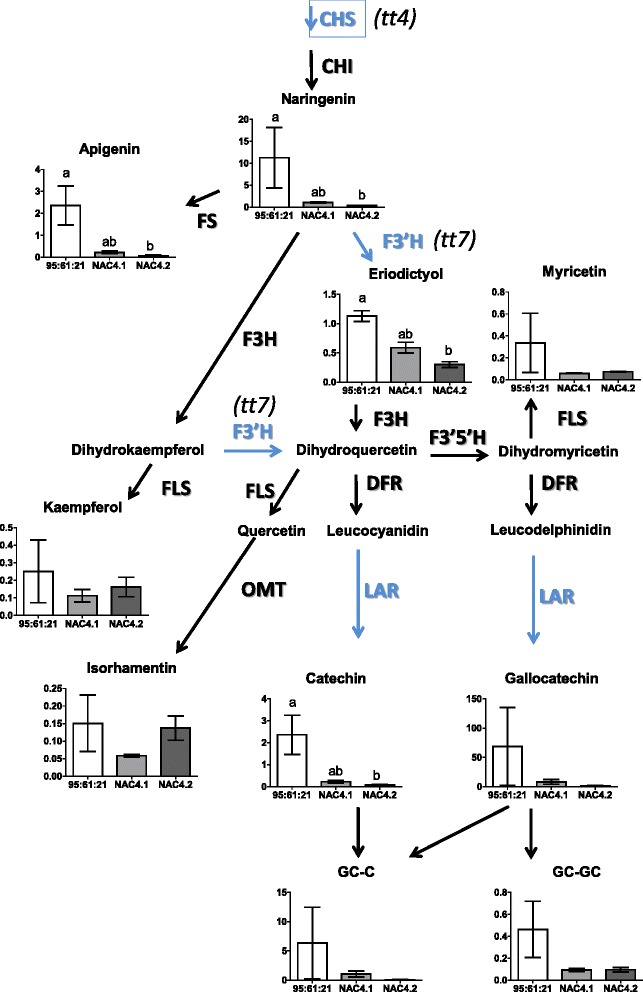
Down-regulation of flavan-3-ol in *PaNAC03* OE-lines. Quantification of flavonoids in the WT line 95:61:21 and the *PaNAC03* OE-lines 4.1 and 4.2 after two weeks culture. The flavonoids were quantified according to Hammerbacher et al. (2014) (*N =* 3) superscript letters indicate significant differences (Kruskal-Wallis test)

Given the concomitant down-regulation of Norway spruce homologs to key genes in the flavonoid pathway, we analysed the levels of specific specialized metabolites in the *PaNAC03* OE-lines namely of the major stilbenes, the immediate catalytic products of *Pa*LAR3, catechin and gallocatechin, and finally a number of flavonoids. The major stilbene in Norway spruce, astringin, showed no significant differences between the WT and the *PaNAC03* OE-lines, neither did the flavonoids kaempferol or isorhamnetin (Fig. 6). However the levels of naringenin, apigenin, eriodictyol and catechin, gallocatechin and their dimers were all lower in OE-line 4.2 (*P <* 0.05, One way-ANOVA) and line 4.1 (0.1 > P > 0.05, One-way ANOVA) (Fig. 6).

#### *Pa*NAC03 does not suppress the activity of the *PaLAR3* promoter

**Fig. 7:**
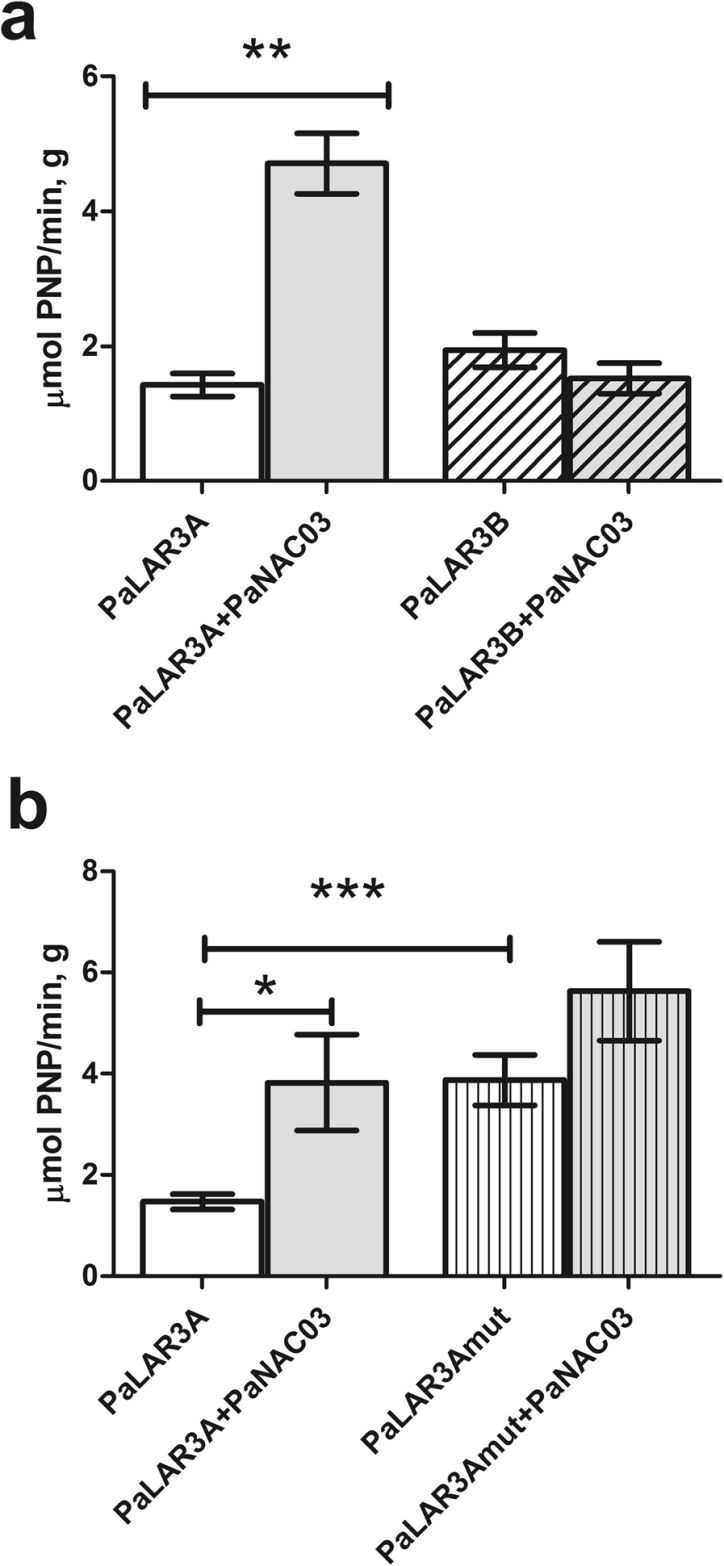
Transactivation of *PaLAR3* promoters by PaNAC03. Figure **a**, transactivation of native *PaLAR3A* (open and light grey bars) and *PaLAR3B* promoters (open and light grey hashed bars) by PaNAC03 in *N. benthamiana*, the figure shows the results from one of three representative experiments (*N =* 12). Figure **b** shows one representative transactivation experiment with native *PaLAR3A* (open and light grey bars) and *PaLAR3A_mut* promoters (open and light striped bars) by PaNAC03 in *N. benthamiana* (*N =* 9). The mean +/− SE is indicated for each measurement. Asterisks indicate significant differences * = *P <* 0.05; ** = *P <* 0.01; *** = *P <* 0.001 (Mann–Whitney *U*-test)

One of the consistently down regulated genes (*PaLAR3*, MA_10001337g0010) has been thoroughly studied before [47, 56] and the promoters from two different alleles, *PaLAR3A* and *PaLAR3B,* have been isolated, the promoters show a high over all similarity and they differ primarily by two indel-regions present in the *PaLAR3A* promoter only, containing two putative NAC binding sites (Nemesio-Gorriz 2016). The WT line, 95:61:21, used in this experiment is homozygous for the *PaLAR3A* allele (data not shown), thus we hypothesized that PaNAC03 repressed *PaLAR3A* (MA_10001337g0010), and possibly also MA_10359605g0010 and MA_10434709g0010, by direct interaction with the promoter of these genes. To test this hypothesis, the *PaLAR3A* and *PaLAR3B* promoters were cloned into a *GUS* reporter vector and were used in a transactivation experiment in *N. bethamiana* leaves with and without *Pa*NAC03, the basal activity of the promoters and effect of PaNAC03 on these promoters were quantified. The basal expression of the *PaLAR3A* and *PaLAR3B* promoters was similar (Figure7a). However, co-expression of PaNAC03 strongly activated the *PaLAR3A* promoter (*P <* 0.05) but did not affect the activity of the *PaLAR3B* promoter (Fig. 7a), showing a different interaction of PaNAC03 with the two promoters.

To investigate if the two putative NAC binding sites in the indel-region unique to the *PaLAR3A* promoter (Additional file 3) were the targets for PaNAC03 causing the specific activation of the *PaLAR3A* promoter we mutated these sites constructing promoter p*PaLAR3A_mut.* The mutated promoter was cloned into the *GUS* reporter vector. Thereafter *PaNAC03* was co-expressed with either *pPaLAR3A_mut* or the native *pPaLAR3A* in *N. bethamiana* leaves. Interestingly the basal activity of *pPaLAR3A_mut* was higher than that of the native *PaLAR3A* promoter (Fig. 7b) showing that these putative NAC binding sites can affect *PaLAR3A* expression, the deletion of the NAC binding sites did however not affect the transactivation of *pPaLAR3A* by *Pa*NAC03, there was no significant difference in relative activity of *pPaLAR3A_mut* (1.5 +/− 0.8 times the promoter alone) or the native *pPaLAR3A* (2.6 +/− 1.8 times the promoter alone).

## Discussion

In this study we identified seven gene models in the Norway spruce genome assembly v 1.0 that show homology to the stress-induced subgroup III-3 NACs in *Arabidopsis* [1]. Generally, the Norway spruce subgroup III-3 NAC gene family members are highly similar displaying the conserved N-terminal A-E motifs characterizing NAC domain proteins [22] and also a relatively conserved C-terminal region including a conserved C-terminal motif SEKEE (V/I) QSSFRLE, a motif present also in most sequences amplified with the marker Sb29 [57]. The conserved C-terminal region in Norway spruce subgroup III-3 members is different from the C-terminal motifs *Arabidopsis* subgroup III-3 members and likely the Norway spruce members do not have a transactivation domain similar to the *Arabidopsis* members [1], and it is unclear if the function of Norway spruce subgroup III-3 NAC is similar to that of *Arabidopsis* subgroup III-3 members. The Norway spruce subgroup III-3 NACs share, at least partly, an element of stress inducibility with the *Arabidopsis* members based on the expression data available in the Norway spruce genome portal. Three of the Norway spruce subgroup III-3 gene models were identical to the PUTs *PaNAC03, PaNAC04* and *PaNAC05* from a *de novo* transcriptome assembly of the interaction between Norway spruce and *H. annosum* s.s. [9, 26]. A fourth gene model, MA_86256p0010, showed similarity to another PUT. Taken together, it shows that certain Norway spruce subgroup III-3 NACs, like their *Arabidopsis* orthologs, respond to biotic stress. Interestingly, the biotic stress responsive gene models to (PaNAC03, PaNAC04, PaNAC05 and MA_86256p0010) cluster together in the phylogeny with MA_103386p0010 and MA_64687p0010, which do not respond to *H. annosum* s.l. inoculation, forming a sister group to the *Arabidopsis* ANAC032, ATAF1, ATAF2 and ANAC102 proteins and to a group of rice NAC genes known to respond to abiotic stress [58]. This differentiation in terms of expression pattern between Norway spruce paralogs is consistent with the concept of subfunctionalization [59].

To confirm the responsiveness to biotic and abiotic stress, the expression of *PaNAC03* and *PaNAC04* was analysed in response to *H. annosum* s.s. infection or wounding in phloem of mature Norway spruce trees by qRT-PCR. Both genes showed significant induction in response to either treatment, but as previously reported for other Norway spruce genes, [5, 7, 9, 60], the induction was higher after inoculation than after wounding. However only *PaNAC03* transcription was significantly induced in response to *H. parviporum* treatment. This discrepancy seen in the induction patterns of *PaNAC04* between experiments could be an effect of several different factors; obviously different organs of conifers show different transcriptional responses to pathogens in seedlings, the responses appears to be more organ-specific than pathogen-specific [4] suggesting that the organ analysed, phloem versus seedling roots, could explain the differential induction between *PaNAC03* and *PaNAC04.* Furthermore, the age of the host is another factor which may affect the manifested defence responses in conifers. The two fungal species, *H. annosum* s.s. and *H. parviporum,* are known to have different host preferences (as reviewed in [11]) and different capacity to produce phytotoxins [61], suggesting that their interaction with the host may differ and could lead to a differential regulation of *PaNAC04*. However, in our previous studies confronting Norway spruce plants of different ages with *H. parviporum* and *H. annosum* s.s. [5, 7, 9, 60] we have seen consistent induction of defence related genes with the two different fungal species over different age classes and tissues tested. Thus, the gene induction patterns could indicate that *PaNAC03* is likely to have a closer association with the transcriptional responses to biotic stress in Norway spruce than *PaNAC04.* Therefore we selected *PaNAC03* for functional analysis by overexpression in somatic embryogenic cultures.

The overall plan of embryo development is similar in angiosperms and gymnosperms despite their separation approximately 300 million years ago. There are, however, several distinct differences in the embryo development programme between the two plant lineages. In angiosperms the first tissue to differentiate during embryogenesis is the protoderm which is formed by periclinal divisions of cells of the early globular embryo [62]. The formation of the protoderm, which restricts cell expansion, is essential for the remaining developmental process [63]. In contrast, in gymnosperms the surface layer of the embryonal mass divides both periclinally and anticlinally. Nevertheless, the outer cell layer in the embryonal mass in Norway spruce embryos defines a functional protoderm [63, 64]. The developing embryonal masses in the *PaNAC03* OE-lines masses appeared to lack the normal conifer “protoderm” i.e. a smooth outer surface, the ruggedness of the embryo surface were reminiscent of the phenotype of other transgenic Norway spruce lines with a disturbed protoderm formation [35, 63, 64]. The embryonal masses did generally not develop into mature cotelydonary embryos. A small number of mature, but aberrant looking, embryos were recovered from the OE-lines. These embryos showed a normal germination response but a significantly smaller fraction of the germinated embryos showed epicotyl formation and growth. Based on these observations we concluded that the embryo development programme is disturbed at a very early stage in the *PaNAC03* OE-lines.

Among the 482 consistently misregulated gene models identified by transcriptome sequencing of the two *PaNAC03* OE-lines we found a number of genes known to control various aspects of patterning and embryo development in Norway spruce. The most strongly induced gene model is MA_122121g0010 which encodes a HD-ZIP IV protein highly similar to *Pa*HB2 [53] and the Arabidopsis genes *GLABRA 2* and *ANTHOCYANINLESS2* [54, 55, 65] associated with patterning in Norway spruce and Arabidopsis while *PaHB1,* controlling protoderm formation [64]*,* was slightly down regulated in the OE lines. *Pa*HB2 is not expressed during early embryo development in WT-lines [53] and neither is MA_122121g0010. Thus, the misregulation of MA_122121g0010 may be the cause of the aberrant embryo morphology in the OE-lines. Other strongly upregulated gene models in the OE-lines with potential to cause of the aberrant embryo morphology encode *HBK4* (MA_114226g0010), *PaPIN1* (MA_100472g0010) and *PaACT4* (MA_135063g0010), which all have been shown to control Norway spruce somatic embryo development [18, 66–68], and specifically the differentiation of the shoot apical meristem and cotyledons [18, 69] processes which appears to be affected in the *PaNAC03* OE lines. A gene model (MA_10251997g0010) with similarity to the Arabidopsis gene *KANADI1* (AT5G16560.1) shows a three-fold lower expression in the *PaNAC03* OE-lines. In Arabidopsis embryos *KANADI1* is initially expressed in the central domain protoderm at the late globular embryo stage and appears to have a role in specifying the peripheral identity in the developing Arabidopsis embryo in interplay with HD-ZIP III proteins [70–72]. It may be noteworthy that, as mentioned before, *PaPIN1* and also a Norway spruce HD-Zip III gene model (MA_10427484g0010) with similarity to ATHB15 (AT1G52150) and appears to show contrasting induction levels compared to the *KANADI*-like gene in OE-lines; these expression patterns are reminiscent of the interaction between the KANADI1, HD-ZIP III and PIN1 in *Arabidopsis* [71, 73]. Taken together the maturation and RNA-seq data indicates that ectopic expression of *PaNAC03* interferes with the protoderm formation and early embryo patterning through misregulation of transcriptional modules controlling these processes. This pleiotropic effect must be taken into consideration when further examining the consistently misregulated genes in *PaNAC03* OE-lines as any gene misregulation may be an effect of the aberrant early embryo development.

About two thirds of the consistently misregulated genes were consistently repressed in the overexpression lines. The consistently repressed genes more commonly associated GO terms related to response to abiotic stimuli, stress responses and responses to hydrogen peroxide. There were three consistently down regulated class III peroxidases, including *PaPrx01*, among the consistently misregulated genes. Previously, *PaPrx01* has been shown to respond to *H. parviporum* treatments in Norway spruce cultures [74] and it is suggested to contribute to H_2_O_2_ production in suspension cultures of Norway spruce, indicating a potential role of PaNAC03 in redox homeostasis under stress in Norway spruce as *H. parviporum* treatments in Norway spruce cultures appears to repress several peroxidases [74].

We observed consistent misregulation of three key genes in the flavonoid biosynthesis pathway in the overexpression lines, a *CHS* homologous to the *Arabidopsis thaliana* gene *transparent testa 4* (*TT4*, AT5G13930), one *F3’H* homologus to the *Arabidopsis* gene *transparent testa 7* (*TT7*, AT5G07990) and the previously described *PaLAR3* gene [47]. Variation in the *PaLAR3* locus associated with enhanced resistance to *H. parviporum* and with increased accumulation of the catalytic product of the enzyme (+) catechin [56].

The concomitant misregulation of key genes in the flavan-3-ol pathway is associated with reduced levels of flavan-3-ols in the OE-lines; both naringenin and apigenin, which are products formed downstream of CHS but before steps catalysed by either F3’H or *Pa*LAR3 were down regulated in the *Pa*NAC03 overexpression lines (as indicated in Fig. 6). Eriodictyol, a catalytic product of F3’H was also reduced. The catalytic product of *Pa*LAR3, (+)-catechin, was also significantly reduced in the OE lines. While other metabolites not directly associated with flavan-3-ol production accumulated to the same levels as in the WT line, showing that the down regulation of key members in the flavan-3-ol pathway lead to a specific reduction in these compounds. Although regulation of anthocyanin or proanthocyanin pathways by NAC TFs is not commonly reported in literature. The NAC TFs *BL,* controlling the blood red flesh phenotype in peach, and ANAC078 in *Arabidopsis* appear to control certain members of the anthocyanin or proanthocyanin pathways [75, 76]. The metabolite and transcriptome profiling of the OE lines appeared to indicate that *Pa*NAC03 could act as a negative regulator of 3-flavanol production in Norway spruce, possibly by acting directly on the misregulated flavonoid biosynthesis genes. To test this possibility we co-expressed *PaNAC03* with the promoter of either of the two alleles at the *PaLAR3* locus [56] in *N. bethamiana* leaves; hypothesising that *Pa*NAC03 would reduce *PaLAR3* promoter activity if it acts as a repressor. However, in this system *Pa*NAC03 strongly activated the promoter of the *PaLAR3A* allele suggesting that *Pa*NAC03 does not act as a negative regulator of flavan-3-ol production by direct interaction with *PaLAR3.* However, the down-regulation of *CHS* transcription*,* encoding the rate-limiting step in flavonoid biosynthesis [77] might have had an effect on substrate availability for downstream metabolite biosynthesis, explaining the lower transcriptional and metabolite levels observed in our study. Transcript profiling also showed an up-regulation of an isoflavone reductase gene that could be involved in lignin biosynthesis [78]. Lignan and lignin biosynthesis directly compete for substrates used in the flavonoid pathway and might therefore also negatively regulate flavonoid biosynthesis, as has been observed in our *Pa*NAC03 over-expressing lines. It is possible that the down-regulation of *CHS, F3’H* and *PaLAR3* genes in *PaNAC03* overexpressing lines could be mediated by another factor such as misregulation of an upstream regulatory gene or the interference of constitutive *PaNAC03* expression with early embryo patterning. It should be noted that flavanols and the *transparent testa* mutants has been linked to auxin homeostasis and polar auxin transport [79, 80] in plants. Another possible explanation to the discrepancy between the overexpression and transactivation experiments is that *Pa*NAC03 act in a heterodimer, as has been shown for other stress-responsive NACs [81, 82], with a currently unidentified TF to downregulate the *CHS, F3’H* and *PaLAR3* genes in the OE-lines. This possibility could be tested by yeast two-hybrid screening of cDNA libraries from embryogenic cultures using *Pa*NAC03 as a bait.

## Conclusion

*PaNAC03* and its orthologs form a sister group to well characterized stress-related angiosperm NAC genes and at least *PaNAC03* is responsive to biotic stress and appear to act in the control of defence associated secondary metabolite production. However, the unexpected embryo phenotype of the *PaNAC03* OE lines emphasizes the still enigmatic connection between specialized metabolism and patterning in plants, raising questions on the role of subgroup III-3 NAC TFs in development and embryo patterning.

## Additional files

Additional file 1: Sequences of primers used in the study. (XLSX 11 kb)
Additional file 2: Scripts used for Nesoni, tophat, cufflinks and cuffdiff. (XLSX 14 kb)
Additional file 3: Figure S2. Schematic representation of the *PaLAR3A* promoter (Genbank accession no. KX574229.1) in black and the *PaLAR3B* promoter (KX574230.1) in red, the white regions in *PaLAR3B* promoter corresponds to deletions in the sequence compared to *PaLAR3A* promoter. The two NAC binding sites (TTTCGT) present in the region unique to the *PaLAR3A* promoter are indicated in yellow. In the *PaLAR3A_mut* promoter it is only these two sites which has been mutated in the remainder of the *PaLAR3A* promoter is intact. (XLSX 69 kb)
Additional file 4: Clustal W Alignment of Norway spruce subgroup III-3 NAC proteins. The coloured boxes correspond to the conserved N-terminal motifs A (light blue), B (pale green), C (pale red, D (lilac) and E (pale gold). The shaded residues indicate residues conserved in the C-terminal region. (XLSX 21 kb)
Additional file 5: Amino acid identity and similarity in subgroup III-3 NAC proteins. Percent amino acid identity (above the diagonal) and similarity (below the diagonal) in the complete protein sequences (A) or the C-terminal part of the proteins (B). (DOCX 16 kb)
Additional file 6: Relative expression of putative *PaNAC3* overexpression lines. The relative expression was determined in relation to the untransformed wild type line 95:61:21. (DOCX 53.1 kb)
Additional file 7: Transcriptional regulation of *PaNAC3* in response to standard maturation treatment in the wild type line 95:61:21. (PDF 447 kb)
Additional file 8: Table S7. RNAseq metrics after Nesoni filtering. (DOCX 19 kb)
Additional file 9: Alignment summaries from tophat. (DOCX 11 kb)
Additional file 10: Enriched GO terms among consistently down- or up-regulated genes in *PaNAC3* overexpression lines. (TIF 45020 kb)
Additional file 11: Consistently up-regulated genes in *PaNAC3* overexpression lines. (DOCX 15 kb)
Additional file 12: Consistently down-regulated genes in *PaNAC3* overexpression lines. (DOCX 17 kb)

## References

[1] Jensen MK, Kjaersgaard T, Nielsen MM, Galberg P, Petersen K, O’Shea C, Skriver K (2010). The Arabidopsis thaliana NAC transcription factor family: structure-function relationships and determinants of ANAC019 stress signalling. Biochem J.

[2] Zhu T, Nevo E, Sun D, Peng J (2012). Phylogenetic analyses unravel the evolutionary history of nac proteins in plants. Evol Int J org Evol.

[3] Nystedt B, Street NR, Wetterbom A, Zuccolo A, Lin Y-C, Scofield DG, Vezzi F, Delhomme N, Giacomello S, Alexeyenko A (2013). The Norway spruce genome sequence and conifer genome evolution. Nature.

[4] Adomas A, Asiegbu FO (2006). Analysis of organ-specific responses of *Pinus sylvestris* to shoot (Gremmeniella abietina) and root (Heterobasidion annosum) pathogens. Physiol Mol Plant Pathol.

[5] Arnerup J, Lind M, Olson A, Stenlid J, Elfstrand M (2011). The pathogenic white-rot fungus Heterobasidion parviporum triggers non-specific defence responses in the bark of Norway spruce. Tree Physiol.

[6] Fossdal CG, Nagy NE, Hietala AM, Kvaalen H, Slimestad R, Woodward S, Solheim H (2012). Indications of heightened constitutive or primed host response affecting the lignin pathway transcripts and phenolics in mature Norway spruce clones. Tree Physiol.

[7] Arnerup J, Nemesio-Gorriz M, Lundén K, Asiegbu FO, Stenlid J, Elfstrand M (2013). The primary module in Norway spruce defence signalling against H. annosum s.l. seems to be jasmonate-mediated signalling without antagonism of salicylate-mediated signalling. Planta.

[8] Yaqoob N, Yakovlev IA, Krokene P, Kvaalen H, Solheim H, Fossdal CG (2012). Defence-related gene expression in bark and sapwood of Norway spruce in response to Heterobasidion parviporum and methyl jasmonate. Physiol Mol Plant Pathol.

[9] Danielsson M, Lunden K, Elfstrand M, Hu J, Zhao T, Arnerup J, Ihrmark K, Swedjemark G, Borg-Karlson AK, Stenlid J (2011). Chemical and transcriptional responses of Norway spruce genotypes with different susceptibility to Heterobasidion spp. infection. BMC Plant Biol.

[10] Davies TJ, Barraclough TG, Chase MW, Soltis PS, Soltis DE, Savolainen V (2004). Darwin’s abominable mystery: Insights from a supertree of the angiosperms. Proc Natl Acad Sci U S A.

[11] Dalman K, Olson A, Stenlid J (2010). Evolutionary history of the conifer root rot fungus Heterobasidion annosum sensu lato. Mol Ecol.

[12] Otrosina WJ, Garbelotto M (2010). Heterobasidion occidentale sp nov and Heterobasidion irregulare nom. nov.: A disposition of North American Heterobasidion biological species. Fungal Biol.

[13] Olsen AN, Ernst HA, Leggio LL, Skriver K (2005). NAC transcription factors: structurally distinct, functionally diverse. Trends Plant Sci.

[14] Yamaguchi M, Demura T (2010). Transcriptional regulation of secondary wall formation controlled by NAC domain proteins. Plant Biotechnol.

[15] Kubo M, Udagawa M, Nishikubo N, Horiguchi G, Yamaguchi M, Ito J, Mimura T, Fukuda H, Demura T (2005). Transcription switches for protoxylem and metaxylem vessel formation. Genes Dev.

[16] Takada S, Hibara K, Ishida T, Tasaka M (2001). The CUP-SHAPED COTYLEDON1 gene of Arabidopsis regulates shoot apical meristem formation. Dev (Cambridge, England).

[17] Duval I, Lachance D, Giguere I, Bomal C, Morency MJ, Pelletier G, Boyle B, MacKay JJ, Seguin A (2014). Large-scale screening of transcription factor-promoter interactions in spruce reveals a transcriptional network involved in vascular development. J Exp Bot.

[18] Larsson E, Sundstrom JF, Sitbon F, von Arnold S (2012). Expression of PaNAC01, a Picea abies CUP-SHAPED COTYLEDON orthologue, is regulated by polar auxin transport and associated with differentiation of the shoot apical meristem and formation of separated cotyledons. Ann Bot.

[19] Puranik S, Sahu PP, Srivastava PS, Prasad M (2012). NAC proteins: regulation and role in stress tolerance. Trends Plant Sci.

[20] Wu Y, Deng Z, Lai J, Zhang Y, Yang C, Yin B, Zhao Q, Zhang L, Li Y, Yang C (2009). Dual function of Arabidopsis ATAF1 in abiotic and biotic stress responses. Cell Res.

[21] Bu Q, Jiang H, Li CB, Zhai Q, Zhang J, Wu X, Sun J, Xie Q, Li C (2008). Role of the Arabidopsis thaliana NAC transcription factors ANAC019 and ANAC055 in regulating jasmonic acid-signaled defense responses. Cell Res.

[22] Ooka H, Satoh K, Doi K, Nagata T, Otomo Y, Murakami K, Matsubara K, Osato N, Kawai J, Carninci P (2003). Comprehensive analysis of NAC family genes in Oryza sativa and Arabidopsis thaliana. DNA Res Int J rapid publ Rep Genes and Genomes.

[23] Jensen MK, Kjaersgaard T, Petersen K, Skriver K (2010). NAC genes: time-specific regulators of hormonal signaling in Arabidopsis. Plant Signal Behav.

[24] Wang X, Basnayake BM, Zhang H, Li G, Li W, Virk N, Mengiste T, Song F (2009). The Arabidopsis ATAF1, a NAC transcription factor, is a negative regulator of defense responses against necrotrophic fungal and bacterial pathogens. Mol Plant-Microbe interactions : MPMI.

[25] Delessert C, Kazan K, Wilson IW, Van Der Straeten D, Manners J, Dennis ES, Dolferus R (2005). The transcription factor ATAF2 represses the expression of pathogenesis-related genes in Arabidopsis. Plant JCell Mol Biol.

[26] Lunden K, Danielsson M, Durling MB, Ihrmark K, Nemesio-Gorriz M, Stenlid J, Asiegbu FO, Elfstrand M. Transcriptional Responses Associated with Virulence and Defence in the Interaction between Heterobasidion annosum s. s. and Norway Spruce. Plos One 2015;10(7):e0131182. 10.1371/journal.pone.0131182 PMC4495060 26151363

[27] Altschul SF, Madden TL, Schaffer AA, Zhang JH, Zhang Z, Miller W, Lipman DJ (1997). Gapped BLAST and PSI-BLAST: a new generation of protein database search programs. Nucleic Acids Res.

[28] Tamura K, Peterson D, Peterson N, Stecher G, Nei M, Kumar S. MEGA5: molecular evolutionary genetics analysis using maximum likelihood, evolutionary distance, and maximum parsimony methods. Molecular Biology and Evolution 2011;28(10):2731–39. 10.1093/molbev/msr121 PMC3203626 21546353

[29] Rice P, Longden I, Bleasby A (2000). EMBOSS: The European Molecular Biology Open Software Suite. Trends Genet.

[30] Stothard P (2000). The sequence manipulation suite: JavaScript programs for analyzing and formatting protein and DNA sequences. Biotechniques.

[31] Karlsson B, Högberg KA (1998). Genotypic parameters and clone x site interaction in clone tests of Norway spruce (Picea abies (L.) Karst.). For Genet.

[32] Stenlid J, Karlsson J-O (1991). Partial intersterility in Heterobasidion annosum. Mycol Res.

[33] Chang S, Puryear J, Cairney J (1993). A simple and efficient method for extracting RNA from pine trees. Plant Mol Biol Report.

[34] Ingestad T, Kähr M (1985). Nutrition and growth of coniferous seedlings at varied relative nitrogen addition rate. Physiol Plant.

[35] Zhu T, Moschou PN, Alvarez JM, Sohlberg JJ, von Arnold S (2016). WUSCHEL-RELATED HOMEOBOX 2 is important for protoderm and suspensor development in the gymnosperm Norway spruce. BMC Plant Biol.

[36] Pfaffl MW, Horgan GW, Dempfle L (2002). Relative expression software tool (REST (c)) for group-wise comparison and statistical analysis of relative expression results in real-time PCR. Nucleic Acids Research.

[37] Livak KJ, Schmittgen TD (2001). Analysis of relative gene expression data using real-time quantitative PCR and the 2(T) (−Delta Delta C) method. Methods.

[38] Vestman D, Larsson E, Uddenberg D, Cairney J, Clapham D, Sundberg E, von Arnold S (2011). Important processes during differentiation and early development of somatic embryos of Norway spruce as revealed by changes in global gene expression. Tree Genet Genomes.

[39] Palovaara J, Hakman I (2008). Conifer WOX-related homeodomain transcription factors, developmental consideration and expression dynamic of WOX2 during *Picea abies* somatic embryogenesis. Plant Mol Biol.

[40] Pfaffl MW, Tichopad A, Prgomet C, Neuvians TP (2004). Determination of stable housekeeping genes, differentially regulated target genes and sample integrity: BestKeeper – Excel-based tool using pair-wise correlations. Biotechnol Lett.

[41] Curtis MD, Grossniklaus U (2003). A gateway cloning vector set for high-throughput functional analysis of genes in planta. Plant Physiol.

[42] Minina EA, Filonova LH, Fukada K, Savenkov EI, Gogvadze V, Clapham D, Sanchez-Vera V, Suarez MF, Zhivotovsky B, Daniel G (2013). Autophagy and metacaspase determine the mode of cell death in plants. J Cell Biol.

[43] Bozhkov PV, von Arnold S (1998). Polyethylene glycol promotes maturation but inhibits further development of Picea abies somatic embryos. Physiol Plant.

[44] Filonova LH, Bozhkov PV, von Arnold S (2000). Developmental pathway of somatic embryogenesis in Picea abies as revealed by time-lapse tracking. J Exp Bot.

[45] Trapnell C, Roberts A, Goff L, Pertea G, Kim D, Kelley DR, Pimentel H, Salzberg SL, Rinn JL, Pachter L (2012). Differential gene and transcript expression analysis of RNA-seq experiments with TopHat and Cufflinks. Nat Protoc.

[46] Trapnell C, Hendrickson DG, Sauvageau M, Goff L, Rinn JL, Pachter L (2013). Differential analysis of gene regulation at transcript resolution with RNA-seq. Nat Biotechnol.

[47] Hammerbacher A, Paetz C, Wright LP, Fischer TC, Bohlmann J, Davis AJ, Fenning TM, Gershenzon J, Schmidt A (2014). Flavan-3-ols in Norway Spruce: Biosynthesis, Accumulation, and Function in Response to Attack by the Bark Beetle-Associated Fungus Ceratocystis polonica1 C W OPEN. Plant Physiol.

[48] An G (1987). Binary Ti vectors for plant transformation and promoter analysis. Methods Enzymol Recombinant DNA.

[49] Gomez-Eichelmann MC, Lark KG (1977). Endo R DpnI restriction of Escherichia coli DNA synthesized in vitro. Evidence that the ends of Okazaki pieces are determined by template deoxynucleotide sequence. J Mol Biol.

[50] Chen H, Nelson RS, Sherwood JL (1994). Enhanced recovery of transformants of Agrobacterium tumefaciens after freeze-thaw transformation and drug selection. Biotechniques.

[51] Wilson KJ, Hughes SG, Jefferson RA, Gallagher SR (1992). The Escherichia coli gus operon: induction and expression of the gus operon in E. coli and the occurrence and use of GUS in other bacteria. GUS protocols Using the GUS gene as reporter of gene expression.

[52] Bradford MM (1976). A rapid and sensitive method for the quantitation of microgram quantities of protein utilizing the principle of protein-dye binding. Anal Biochem.

[53] Ingouff M, Farbos I, Wiweger M, von Arnold S (2003). The molecular characterization of PaHB2, a homeobox gene of the HD-GL2 family expressed during embryo development in Norway spruce. J Exp Bot.

[54] Di Cristina M, Sessa G, Dolan L, Linstead P, Baima S, Ruberti I, Morelli G (1996). The Arabidopsis Athb-10 (GLABRA2) is an HD-Zip protein required for regulation of root hair development. Plant J Cell Mol Biol.

[55] Masucci JD, Rerie WG, Foreman DR, Zhang M, Galway ME, Marks MD, Schiefelbein JW (1996). The homeobox gene GLABRA2 is required for position-dependent cell differentiation in the root epidermis of Arabidopsis thaliana. Dev (Cambridge, England).

[56] Nemesio Gorriz M, Hammerbacher A, Ihrmark K, Kallman T, Olson A, Lascoux M, Stenlid J, Gershenzon J, Elfstrand M (2016). Different alleles of a gene encoding leucoanthocyanidin reductase (PaLAR3) influence resistance against the fungus Heterobasidion parviporum in Picea abies. Plant Physiol.

[57] Perry DJ, Bousquet J (1998). Sequence-tagged-site (sts) markers of arbitrary genes: development, characterization and analysis of linkage in black spruce. Genetics.

[58] García-Morales S, Gómez-Merino FC, Trejo-Téllez LI (2014). NAC transcription factor expression, amino acid concentration and growth of elite rice cultivars upon salt stress. Acta Physiol Plant.

[59] Prince VE, Pickett FB (2002). Splitting pairs: the diverging fates of duplicated genes. Nat Rev Genet.

[60] Oliva J, Rommel S, Fossdal CG, Hietala AM, Nemesio-Gorriz M, Solheim H, Elfstrand M (2015). Transcriptional responses of Norway spruce (Picea abies) inner sapwood against Heterobasidion parviporum. Tree Physiol.

[61] Hansson D, Wubshet S, Olson A, Karlsson M, Staerk D, Broberg A (2014). Secondary metabolite comparison of the species within the Heterobasidion annosum s.l. complex. Phytochemistry.

[62] ten Hove CA, Lu K-J, Weijers D (2015). Building a plant: cell fate specification in the early Arabidopsis embryo. Dev (Cambridge, England).

[63] Sabala I, Elfstrand M, Farbos I, Clapham D, von Arnold S (2000). Tissue-specific expression of Pa18, a putative lipid transfer protein gene, during embryo development in Norway spruce (Picea abies). Plant Mol Biol.

[64] Ingouff M, Farbos I, Lagercrantz U, von Arnold S (2001). PAHB1 is an evolutionary conserved HD-GL2 homeobox gene expressed in the protoderm during Norway spruce embryo development. Genesis.

[65] Kubo H, Peeters AJM, Aarts MGM, Pereira A, Koornneef M (1999). Anthocyaninless2, a homeobox gene affecting anthocyanin distribution and root development in arabidopsis. Plant Cell.

[66] Hakman I, Hallberg H, Palovaara J (2009). The polar auxin transport inhibitor NPA impairs embryo morphology and increases the expression of an auxin efflux facilitator protein PIN during Picea abies somatic embryo development. Tree Physiol.

[67] Palovaara J, Hallberg H, Stasolla C, Luit B, Hakman I (2010). Expression of a gymnosperm PIN homologous gene correlates with auxin immunolocalization pattern at cotyledon formation and in demarcation of the procambium during Picea abies somatic embryo development and in seedling tissues. Tree Physiol.

[68] Schwarzerová K, Vondráková Z, Fischer L, Boříková P, Bellinvia E, Eliášová K, Havelková L, Fišerová J, Vágner M, Opatrný Z (2010). The role of actin isoforms in somatic embryogenesis in Norway spruce. BMC Plant Biol.

[69] Larsson E, Sitbon F, von Arnold S (2012). Differential regulation of Knotted1-like genes during establishment of the shoot apical meristem in Norway spruce (Picea abies). Plant Cell Rep.

[70] Huang T, Harrar Y, Lin C, Reinhart B, Newell NR, Talavera-Rauh F, Hokin SA, Barton MK, Kerstetter RA (2014). Arabidopsis kanadi1 acts as a transcriptional repressor by interacting with a specific cis-element and regulates auxin biosynthesis, transport, and signaling in opposition to hd-zipiii factors. Plant Cell.

[71] Izhaki A, Bowman JL (2007). Kanadi and class iii hd-zip gene families regulate embryo patterning and modulate auxin flow during embryogenesis in arabidopsis. Plant Cell.

[72] Kerstetter RA, Bollman K, Taylor RA, Bomblies K, Poethig RS (2001). KANADI regulates organ polarity in Arabidopsis. Nature.

[73] Ilegems M, Douet V, Meylan-Bettex M, Uyttewaal M, Brand L, Bowman JL, Stieger PA (2010). Interplay of auxin, KANADI and Class III HD-ZIP transcription factors in vascular tissue formation. Dev (Cambridge, England).

[74] Kärkönen A, Warinowski T, Teeri T, Simola L, Fry S (2009). On the mechanism of apoplastic H2O2 production during lignin formation and elicitation in cultured spruce cells—peroxidases after elicitation. Planta.

[75] Morishita T, Kojima Y, Maruta T, Nishizawa-Yokoi A, Yabuta Y, Shigeoka S (2009). Arabidopsis nac transcription factor, anac078, regulates flavonoid biosynthesis under high-light. Plant Cell Physiol.

[76] Zhou H, Lin-Wang K, Wang H, Gu C, Dare AP, Espley RV, He H, Allan AC, Han Y (2015). Molecular genetics of blood-fleshed peach reveals activation of anthocyanin biosynthesis by NAC transcription factors. Plant J.

[77] Winkel-Shirley B (2001). Flavonoid biosynthesis. a colorful model for genetics, biochemistry, cell biology, and biotechnology. Plant Physiol.

[78] Dinkova-Kostova AT, Gang DR, Davin LB, Bedgar DL, Chu A, Lewis NG (1996). (+)-Pinoresinol/(+)-lariciresinol reductase from Forsythia intermedia. Protein purification, cDNA cloning, heterologous expression and comparison to isoflavone reductase. J Biol Chem.

[79] Buer CS, Kordbacheh F, Truong TT, Hocart CH, Djordjevic MA (2013). Alteration of flavonoid accumulation patterns in transparent testa mutants disturbs auxin transport, gravity responses, and imparts long-term effects on root and shoot architecture. Planta.

[80] Peer WA, Cheng Y, Murphy AS (2013). Evidence of oxidative attenuation of auxin signalling. J Exp Bot.

[81] Jeong JS, Park YT, Jung H, Park S-H, Kim J-K (2009). Rice NAC proteins act as homodimers and heterodimers. Plant Biotechnology Reports.

[82] Hegedus D, Yu M, Baldwin D, Gruber M, Sharpe A, Parkin I, Whitwill S, Lydiate D (2003). Molecular characterization of Brassica napus NAC domain transcriptional activators induced in response to biotic and abiotic stress. Plant Mol Biol.

