# Supplementary material for: Overexpression of PaNAC03, a stress induced NAC gene family transcription factor in Norway spruce leads to reduced flavonol biosynthesis and aberrant embryo development
Source: BMC Plant Biol. 2017 Jan 6;17:6. doi: 10.1186/s12870-016-0952-8 (PMC5219727; doi:10.1186/s12870-016-0952-8)
Supplement: Additional file 5: — Amino acid identity and similarity in subgroup III-3 NAC proteins. Percent amino acid identity (above the diagonal) and similarity (below the diagonal) in the complete protein sequences (A) or the C-terminal part of the proteins (B). (DOCX 16 kb) [file 12870_2016_952_MOESM5_ESM.docx]

**Supplementary file S2.** Scripts used for Nesoni, tophat, cufflinks and cuffdiff.

# Nesoni

nesoni clip: clipped pairs:/proj/mykopat-panac/panac2/raw-data/Sample_Nac4-1_1/Nac4-1_1_GTGGCC_L004_R1_001.fastq.gz /proj/mykopat-panac/panac2/raw-data/Sample_Nac4-1_1/Nac4-1_1_GTGGCC_L004_R2_001.fastq.gz

# This is a job script to do ... [test nesoni clip] remember the script cannot start with a digit

#$ -M kerstin.dalman@slu.se

# -m bae

# Secon, tell the system that you want to execute the job in the current directory

#$ -cwd

# Request 48 hours, 0 minutes, 0 seconds run time

#$ -l h_rt=48:0:0

# Request 4GB memory (check the need for your specific program!)

#$ -l h_vmem=4G

#Place your commands below this line

module load nesoni

nesoni clip: clipped \

--out-separate yes \

--gzip yes \

pairs: /proj/mykopat-panac/panac2/raw-data/Sample_Nac4-1_1/Nac4-1_1_GTGGCC_L004_R1_001.fastq.gz /proj/mykopat-panac/panac2/raw-data/Sample_Nac4-1_1/Nac4-1_1_GTGGCC_L004_R2_001.fastq.gz

# Bowtie

#This is a script to create a bowtie2 index for Trinity contaminant free

# Request 12 hours, 0 minutes, 0 seconds run time

#$ -l h_rt=12:0:0

# Request 4GB memory (check the need for your specific program!)

#$ -l h_vmem=2G

# Request mails to be sent when the job finishes (e) or if it fails (a)

#$ -m ae

# Run in the current directory (otherwise it will run from your home

# directory)

#$ -cwd

# Keep all output in a single file

#$ -j y

# We like to run bowtie2 on several "cores" at the same time

#$ -pe smp 8

# Place your commands below this line

# Load required modules

module load bowtie2/2.2.4

# Run bowtie2

bowtie2-build /proj/mykopat-pafullsib/genome/Trinity_contaminant_free.tar/Trinity_contaminant_free/Picea-abies_transcripts-in-silico-validated_august-2012.fa Trinity_contaminant_free_index

# Tophat

# This is a job script to align sample 61-21_2a to the Trinity contaminant free Trinity_contaminant_free_index

# Request 12 hours, 0 minutes, 0 seconds run time

#$ -l h_rt=24:0:0

# Request 4GB memory (check the need for your specific program!)

#$ -l h_vmem=2G

# Request mails to be sent when the job finishes (e) or if it fails (a)

#$ -m ae

# Run in the current directory (otherwise it will run from your home directory)

#$ -cwd

# Keep all output in a single file

#$ -j y

# We like to run tophat on several "cores" at the same time

#$ -pe smp 8

# Place your commands below this line

# Load required modules

module load tophat/2.0.13

module load bowtie2/2.2.4

# Create output directory

mkdir th_tr_61-21_2a

# Run tophat (note the backslash wich joins the lines into a single command)

tophat -p 8 -o th_tr_61-21_2a /proj/mykopat-panac/ngs/refgenomes/bowtie_indexed/Trinity_contaminant_free_index \

/proj/mykopat-panac/panac2/ngs/analyzed_read_files/nesoni/61-21_2a/clipped_R1.fq.gz \

/proj/mykopat-panac/panac2/ngs/analyzed_read_files/nesoni/61-21_2a/clipped_R2.fq.gz,/proj/mykopat-panac/panac2/ngs/analyzed_read_files/nesoni/61-21_2a/clipped_single.fq.gz

# Cufflinks

# This is a job script to run cufflinks for sample th_tr_61_21_2a

# Request 12 hours, 0 minutes, 0 seconds run time

#$ -l h_rt=12:0:0

# Request 4GB memory (check the need for your specific program!)

#$ -l h_vmem=1G

# Request mails to be sent when the job finishes (e) or if it fails (a)

#$ -m ae

# Run in the current directory (otherwise it will run from your home

#directory)

#$ -cwd

# Keep all output in a single file

#$ -j y

# We like to run cufflinks on several "cores" at the same time

#$ -pe smp 8

# Place your commands below this line Load required modules

module load cufflinks

# Create output directory

mkdir clout_tr_61-21_2a

# Run cufflinks (note the backslash wich joins the lines into a single

# command)

cufflinks -p 8 -o clout_tr_61-21_2a /proj/mykopat-panac/panac2/ngs/tophat/tophat_trinity/th_tr_61-21_2a/accepted_hits.bam

# Cuffmerge

# This is a job script to run cuffmerge for cufflinks assembly trinity cl_tr_assembly_list.txt

# Request 12 hours, 0 minutes, 0 seconds run time

#$ -l h_rt=12:0:0

# Request 4GB memory (check the need for your specific program!)

#$ -l h_vmem=1G

# Request mails to be sent when the job finishes (e) or if it fails (a)

#$ -m ae

# Run in the current directory (otherwise it will run from your home

#directory)

#$ -cwd

# Keep all output in a single file

#$ -j y

# We like to run cufflinks on several "cores" at the same time

#$ -pe smp 8

# Place your commands below this line Load required modules

module load cufflinks

# Create output directory

mkdir cm_tr

# Run cuffmerge (note the backslash wich joins the lines into a single

# command)

cuffmerge -p 8 -o cm_tr -s /proj/mykopat-panac/ngs/refgenomes/bowtie_indexed/Trinity_contaminant_free/Picea-abies_transcripts-in-silico-validated_august-2012.fasta /proj/mykopat-panac/panac2/ngs/scripts/cufflinks/cl_tr_assembly_list.txt

# Cuffquant

# This is a job script to run cuffquant for Trinity contaminant free, sample 61-21_2a

# Request 48 hours, 0 minutes, 0 seconds run time

#$ -l h_rt=48:0:0

# Request 8GB memory (check the need for your specific program!)

#$ -l h_vmem=8G

# Request mails to be sent when the job finishes (e) or if it fails (a)

#$ -m ae

# Run in the current directory (otherwise it will run from your home directory)

#$ -cwd

# Keep all output in a single file

#$ -j y

# We like to run tophat on several "cores" at the same time

#$ -pe smp 8

# Place your commands below this line

# Load required modules

module load cufflinks

# Create output directory

mkdir cuffq_tr_61-21_2a

# Run cuffquant (note the backslash wich joins the lines into a single command)

cuffquant -p 8 -o cuffq_tr_61-21_2a /proj/mykopat-panac/panac2/ngs/cuffmerge/cuffmerge_trinity/cm_tr/merged.gtf /proj/mykopat-panac/panac2/ngs/tophat/tophat_trinity/th_tr_61-21_2a/accepted_hits.bam

# Cuffdiff

# This is a job script to run cuffdiff for Trinity contaminant free and cxb files from cuffquant

# Request 48 hours, 0 minutes, 0 seconds run time

#$ -l h_rt=48:0:0

# Request 8GB memory (check the need for your specific program!)

#$ -l h_vmem=8G

# Request mails to be sent when the job finishes (e) or if it fails (a)

#$ -m ae

# Run in the current directory (otherwise it will run from your home directory)

#$ -cwd

# Keep all output in a single file

#$ -j y

# We like to run tophat on several "cores" at the same time

#$ -pe smp 8

# Place your commands below this line

# Load required modules

module load cufflinks

# Create output directory

mkdir cuffdiff_tr_u_2

# Run cuffdiff (note the backslash wich joins the lines into a single command)

cuffdiff -p 8 -o cuffdiff_tr_u_2 -L wt,Nac4-1,Nac4-2 -u /proj/mykopat-panac/panac2/ngs/cuffmerge/cuffmerge_trinity/cm_tr/merged.gtf /proj/mykopat-panac/panac2/ngs/cuffquant/cuffquant_trinity/cuffq_tr_61-21_2a/abundances.cxb,/proj/mykopat-panac/panac2/ngs/cuffquant/cuffquant_trinity/cuffq_tr_61-21_2b/abundances.cxb,/proj/mykopat-panac/panac2/ngs/cuffquant/cuffquant_trinity/cuffq_tr_61-21_4/abundances.cxb /proj/mykopat-panac/panac2/ngs/cuffquant/cuffquant_trinity/cuffq_tr_Nac4-1_1/abundances.cxb,/proj/mykopat-panac/panac2/ngs/cuffquant/cuffquant_trinity/cuffq_tr_Nac4-1_3/abundances.cxb,/proj/mykopat-panac/panac2/ngs/cuffquant/cuffquant_trinity/cuffq_tr_Nac4-1_4/abundances.cxb /proj/mykopat-panac/panac2/ngs/cuffquant/cuffquant_trinity/cuffq_tr_Nac4-2_1/abundances.cxb,/proj/mykopat-panac/panac2/ngs/cuffquant/cuffquant_trinity/cuffq_tr_Nac4-2_2/abundances.cxb,/proj/mykopat-panac/panac2/ngs/cuffquant/cuffquant_trinity/cuffq_tr_Nac4-2_3/abundances.cxb
