# Supplementary material for: Overexpression of PaNAC03, a stress induced NAC gene family transcription factor in Norway spruce leads to reduced flavonol biosynthesis and aberrant embryo development
Source: BMC Plant Biol. 2017 Jan 6;17:6. doi: 10.1186/s12870-016-0952-8 (PMC5219727; doi:10.1186/s12870-016-0952-8)
Supplement: Additional file 6: — Relative expression of putative PaNAC3 overexpression lines. The relative expression was determined in relation to the untransformed wild type line 95:61:21. (DOCX 53.1 kb) [file 12870_2016_952_MOESM6_ESM.docx]

**Supplementary file S3. Clustal W Alignment of Norway spruce subgroup III-3 NAC proteins.** The coloured boxes correspond to the conserved N-terminal motifs A (light blue), B (pale green), C (pale red, D (lilac) and E (pale gold). The shaded residues indicate residues conserved in the C-terminal region.
