# Supplementary figures and images for: Overexpression of PaNAC03, a stress induced NAC gene family transcription factor in Norway spruce leads to reduced flavonol biosynthesis and aberrant embryo development
Source: BMC Plant Biol. 2017 Jan 6;17:6. doi: 10.1186/s12870-016-0952-8 (PMC5219727; doi:10.1186/s12870-016-0952-8)

## *PaNAC03*

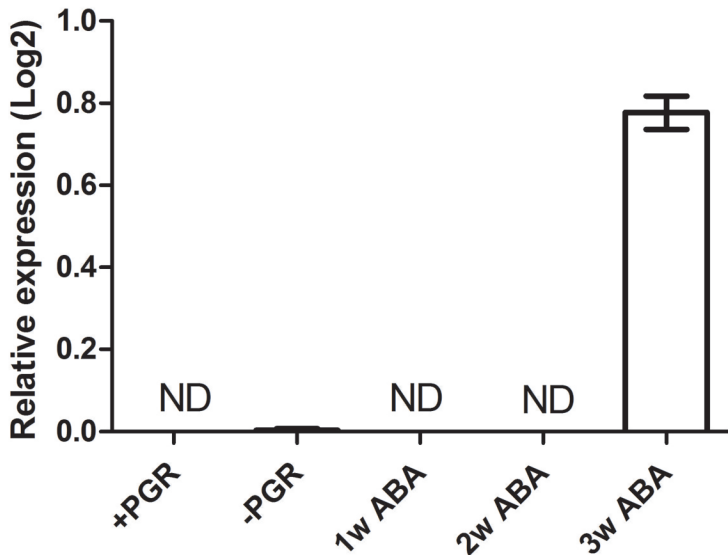

Supplement: Additional file 7: — Transcriptional regulation of PaNAC3 in response to standard maturation treatment in the wild type line 95:61:21. (PDF 447 kb) [file 12870_2016_952_MOESM7_ESM.pdf]

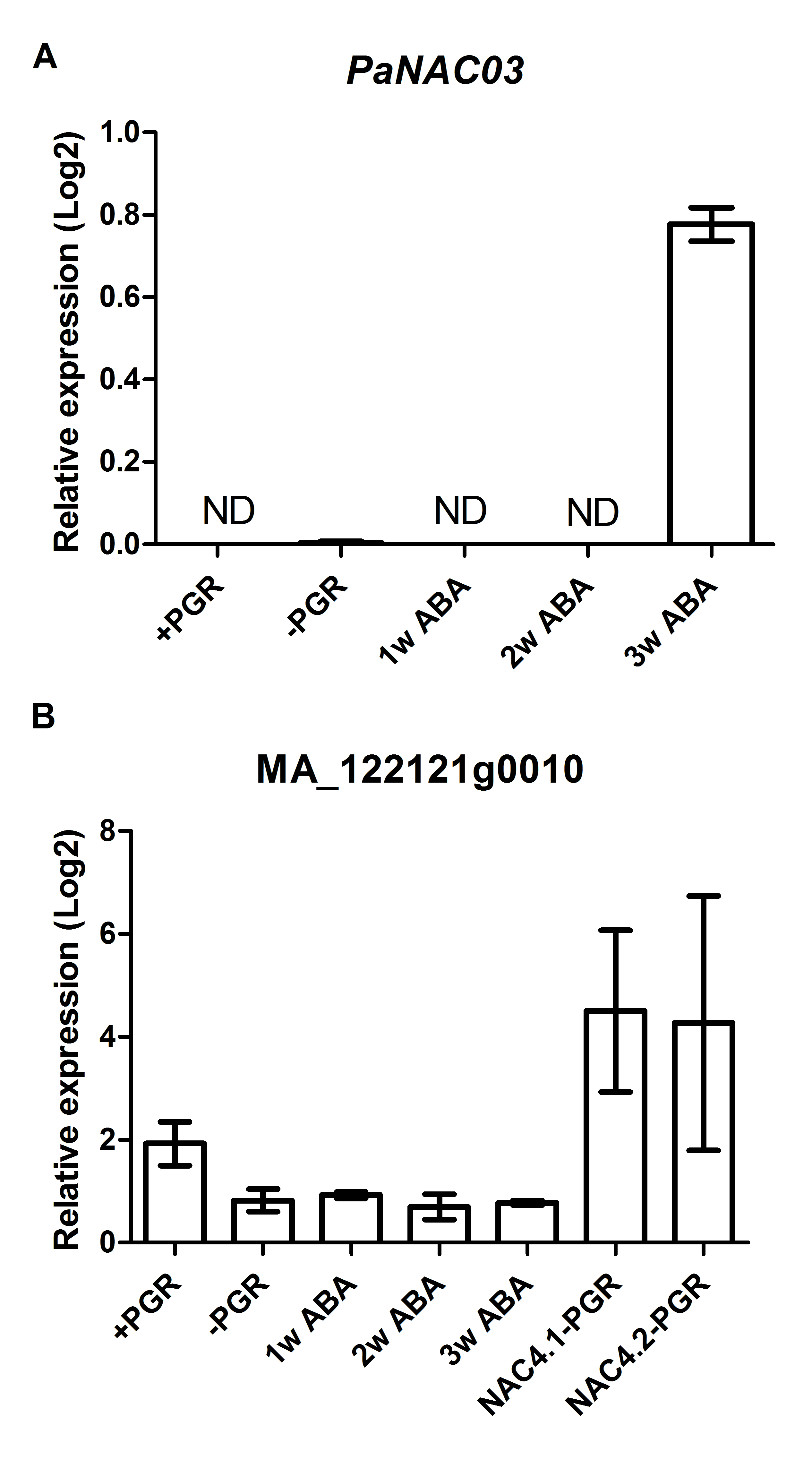

Supplement: Additional file 10: — Enriched GO terms among consistently down- or up-regulated genes in PaNAC3 overexpression lines. (TIF 45020 kb) [file 12870_2016_952_MOESM10_ESM.tif]
