# Supplementary material for: Overexpression of PaNAC03, a stress induced NAC gene family transcription factor in Norway spruce leads to reduced flavonol biosynthesis and aberrant embryo development
Source: BMC Plant Biol. 2017 Jan 6;17:6. doi: 10.1186/s12870-016-0952-8 (PMC5219727; doi:10.1186/s12870-016-0952-8)
Supplement: Additional file 8: Table S7. — RNAseq metrics after Nesoni filtering. (DOCX 19 kb) [file 12870_2016_952_MOESM8_ESM.docx]

**Supplementary file S4.** **Amino acid identity and similarity in subgroup III-3 NAC proteins.** Percent amino acid identity (above the diagonal) and similarity (below the diagonal) in the complete protein sequences (A) or the C-terminal part of the proteins (B).
