# Supplementary material for: Overexpression of PaNAC03, a stress induced NAC gene family transcription factor in Norway spruce leads to reduced flavonol biosynthesis and aberrant embryo development
Source: BMC Plant Biol. 2017 Jan 6;17:6. doi: 10.1186/s12870-016-0952-8 (PMC5219727; doi:10.1186/s12870-016-0952-8)
Supplement: Additional file 9: — Alignment summaries from tophat. (DOCX 11 kb) [file 12870_2016_952_MOESM9_ESM.docx]

**Supplementary file S5**.

| Line | Expression* | Insert verified |
| --- | --- | --- |
| 1.1 | 1.45 | Yes |
| 2.1 | 0.98 | Yes |
| 2.3 | 2.23 | No |
| 2.4 | 0.94 | Yes |
| 2.5 | 0.86 | Yes |
| 2.6 | 1.21 | Yes |
| 3.1 | 0.94 | Yes |
| 4.1 | 1.73 | Yes |
| 4.2 | 1.72 | Yes |
| WT | 1 | - |

*ratio target gene in OE line vs. wild type (corrected for elongation factor alpha)
