# Supplementary material for: Overexpression of PaNAC03, a stress induced NAC gene family transcription factor in Norway spruce leads to reduced flavonol biosynthesis and aberrant embryo development
Source: BMC Plant Biol. 2017 Jan 6;17:6. doi: 10.1186/s12870-016-0952-8 (PMC5219727; doi:10.1186/s12870-016-0952-8)
Supplement: Additional file 11: — Consistently up-regulated genes in PaNAC3 overexpression lines. (DOCX 15 kb) [file 12870_2016_952_MOESM11_ESM.docx]

**Supplementary Table S7. RNAseq metrics after Nesoni filtering**

| Sample | Read-2 too short after quality clip | Read-2 too short after adaptor clip | Read-2 kept | Read-2 average input length | Average output length | Pairs kept after clipping | Reads kept after clipping |
| --- | --- | --- | --- | --- | --- | --- | --- |
| 61_21_2a | 102,601 | 8,493 | 15,692,232 | 124.000 | 117.581 | 15,690,475 | 104,771 |
| 61_21_2b | 113,789 | 6,566 | 16,504,033 | 124.000 | 119.488 | 16,503,081 | 115,033 |
| 61_21_4 | 114,239 | 11,533 | 17,274,102 | 124.000 | 120.390 | 17,273,022 | 115,819 |
| Nac4-1_1 | 106,868 | 5,618 | 16,554,627 | 124.000 | 120.721 | 16,553,769 | 107,968 |
| Nac4-1_3 | 104,273 | 4,1 | 15,919,429 | 124.000 | 120.103 | 15,918,786 | 105,088 |
| Nac4-1_4 | 107,81 | 7,749 | 15,788,748 | 124.000 | 120.342 | 15,787,636 | 109,292 |
| Nac4-2_1 | 116,304 | 38,356 | 15,908,363 | 124.000 | 120.814 | 15,901,869 | 124,457 |
| Nac4-2_2 | 117,64 | 5,939 | 17,821,368 | 124.000 | 120.913 | 17,820,618 | 118,531 |
| Nac4-2_3 | 111,118 | 18,127 | 15,482,847 | 124.000 | 121.332 | 15,479,836 | 114,898 |
