# Supplementary material for: Overexpression of PaNAC03, a stress induced NAC gene family transcription factor in Norway spruce leads to reduced flavonol biosynthesis and aberrant embryo development
Source: BMC Plant Biol. 2017 Jan 6;17:6. doi: 10.1186/s12870-016-0952-8 (PMC5219727; doi:10.1186/s12870-016-0952-8)
Supplement: Additional file 12: — Consistently down-regulated genes in PaNAC3 overexpression lines. (DOCX 17 kb) [file 12870_2016_952_MOESM12_ESM.docx]

**Supplementary file S8. Alignment summaries from tophat**

*of the mapped reads
